# Analytical model of peptide mass cluster centres with applications

**DOI:** 10.1186/1477-5956-4-18

**Published:** 2006-09-23

**Authors:** Witold E Wolski, Malcolm Farrow, Anne-Katrin Emde, Hans Lehrach, Maciej Lalowski, Knut Reinert

**Affiliations:** 1 School of Mathematics and Statistics, Merz Court, University of Newcastle upon Tyne, NE1 7RU, UK; 2 Institute for Computer Science, Free University Berlin, Takustr. 9, 14195 Berlin, Germany; 3 Max Delbrück Center for Molecular Medicine, Robert-Roessle-Str. 10, D-13125 Berlin-Buch, Germany; 4 Max Planck Institute for Molecular Genetics, Ihnestraße 63-73, D-14195 Berlin, Germany

## Abstract

**Background:**

The elemental composition of peptides results in formation of distinct, equidistantly spaced clusters across the mass range. The property of peptide mass clustering is used to calibrate peptide mass lists, to identify and remove non-peptide peaks and for data reduction.

**Results:**

We developed an analytical model of the peptide mass cluster centres. Inputs to the model included, the amino acid frequencies in the sequence database, the average length of the proteins in the database, the cleavage specificity of the proteolytic enzyme used and the cleavage probability. We examined the accuracy of our model by comparing it with the model based on an *in silico *sequence database digest. To identify the crucial parameters we analysed how the cluster centre location depends on the inputs. The distance to the nearest cluster was used to calibrate mass spectrometric peptide peak-lists and to identify non-peptide peaks.

**Conclusion:**

The model introduced here enables us to predict the location of the peptide mass cluster centres. It explains how the location of the cluster centres depends on the input parameters. Fast and efficient calibration and filtering of non-peptide peaks is achieved by a distance measure suggested by Wool and Smilansky.

## Background

The mass spectrometric (MS) technique is widely used to identify proteins in biological samples [1-4]. The proteins are cleaved into peptides by a residue specific protease, *e.g*. trypsin. The resulting cleavage products can then be analysed by Peptide Mass Fingerprinting (PMF) [5] or subjected to MS/MS fragment ion analysis [6,7], which both rely on the comparison of peptide or peptide fragment ion spectra with spectra simulated from protein sequence databases [8].

The sensitivity and specificity of the peptide identification can be increased by various post-processing methods, for example calibration [9-12] and identification of non-peptide peaks [10,13,14]. The fact that peptide masses are not uniformly distributed across the mass range but form equidistantly spaced clusters [15] is employed by some of these methods. In dependence on the atomic composition of the peptide, the monoisotopic mass would emerge below (e.g. cystein rich peptides) or above (e.g. lysine rich peptides) the cluster centres. The deviation from the cluster centre is a result of the mass defect, which is the difference between the nominal mass and the monoisotopic mass (Table 1). The mass defect is a result of atom fusion [16,17].

**Table 1:** Masses of Atoms

	Atom	monoisotopic	nominal	mass defect
1	H	1.00782	1	0.00782
2	C	12.00000	12	0.00000
3	N	14.003074	14	0.003074
4	0	15.99491	16	-0.00032
5	S	31.97207	32	-0.00087

### Calibration

Mass spectrometric peptide peak-lists of peptide mass finger print experiments [18] can be calibrated by comparing the location of measured peptide masses with the location of the peptide mass cluster centres. Gras et al. [19] suggested the use of maximum likelihood methods in order to determine the calibration coefficients *a *and *b*. They defined the likelihood function by:

∑iP(ami+b,Δm),     (1)
 MathType@MTEF@5@5@+=feaafiart1ev1aaatCvAUfKttLearuWrP9MDH5MBPbIqV92AaeXatLxBI9gBaebbnrfifHhDYfgasaacH8akY=wiFfYdH8Gipec8Eeeu0xXdbba9frFj0=OqFfea0dXdd9vqai=hGuQ8kuc9pgc9s8qqaq=dirpe0xb9q8qiLsFr0=vr0=vr0dc8meaabaqaciaacaGaaeqabaqabeGadaaakeaadaaeqbqaaiabdcfaqjabcIcaOiabdggaHjabd2gaTnaaBaaaleaacqWGPbqAaeqaaOGaey4kaSIaemOyaiMaeiilaWIaeyiLdqKaemyBa0MaeiykaKcaleaacqWGPbqAaeqaniabggHiLdGccqGGSaalcaWLjaGaaCzcamaabmGabaGaeGymaedacaGLOaGaayzkaaaaaa@41C6@

where *m*_*i *_is the *i*-th mass in the peak-list, and Δ*m *is a search window. *P*(*m*, Δ*m*) is the probability to find a mass in [*m*, *m *+ Δ*m*] given the theoretical distribution of peptide masses. The parameters *a*, *b *for arg_max _∑_*i *_*P*(*am*_*i *_+ *b*, Δ*m*) can then be used to calibrate the peak-lists. The authors, however, do not provide information on whether *P*(*m*, Δ*m*) was determined from the exact distribution of the peptide masses or if a model approximating the distribution was used. They also do not mention which algorithm was used to maximise the likelihood. They reported that a mass measurement accuracy of 0.2*Da *and better was obtained after calibration.

Wool and Smilansky [10] have used Discrete Fourier Transformation (DFT) to determine the frequency *λ *and phase *ϕ *of a peak-list or mass spectrum. By comparing the experimental *λ *and *ϕ *with the theoretical *λ *= 1.000495 and *ϕ *= 0, they determined the slope and intercept of the calibration function. The authors reported a 40 – 60% reduction of the mass measurement error. Furthermore, they presented a scoring scheme for sequence database searches. This scoring scheme approximates the probability *P*(*m*, Δ*m*) to observe a peptide peak of mass *m *with given measurement error Δ*m*.

### Matrix noise filtration

The most widely used MALDI matrices for the analysis of peptides are 3,5-Dimethoxy-4-hydroxycinnamic acid (*synapic acid*), alpha-Cyano-4-hydroxycinnamic acid (*alpha cyano*) [20] and 2,5-dihydroxybenzoic acid (*DHB*) [21]. Unfortunately, clusters of matrix molecules can be ionised and cause peaks in the same mass range where peptide peaks are measured. Matrix aggregate formation can be minimised but not eliminated by adding ammonium acetate [21].

Some of the database search scoring schemes incorporate the number of signals (peaks) not assigned to a protein when computing the identification scores [22]. Therefore, the presence of matrix signals in MS spectra decreases the sensitivity of the MS spectra interpretation. Hence, the removal of peaks strongly deviating from the cluster centres is applied [21,23]. The measure of deviation from cluster centres introduced here provides a simple tool to filter non-peptide peaks.

### Data reduction

A further application which employs the property of peptide mass clustering is the binning of the mass measurement range. By applying this technique the amount of data is reduced, thus increasing the speed with which the pairwise comparison of spectra can be made [24,25].

All these applications require us to know the exact location of or the distance between the peptide mass cluster centres. The distance between the cluster centres, which we will henceforth call wavelength *λ*, is commonly computed by first generating an *in silico *digest of the database. Afterwards, the linear dependence between the decimal point and the integer part is determined by regression analysis, for a relatively small mass range of 500 to 1000*Da *[23]. Various authors report different values of the distance between clusters: Wool and Smilansky reported 1.000495 [10], Gay et al. 1.000455 [15], while Tabb et al. used a wavelength of 1.00057 [24].

In this work we present an analytical model allowing us to predict the mass of the peptide cluster centres. The parameters of the model include: the frequencies of the amino acids in the sequence database [26], the average protein length of the proteins in the database, the cleavage sites of the proteolytic enzyme and the cleavage probability. Based on this model we introduced a measure of deviation of peptide masses from the nearest cluster centre, which is a refinement of a measure proposed by Wool and Smilansky [10]. Using this distance measure, we developed a calibration procedure which employs least squares linear regression in order to determine the affine model of the mass measurement error and subsequently to calibrate the spectra. Using this method we reached higher calibration accuracy as reported by Wool and Smilansky [10], and Gras et al [19]. We used the same distance measure to identify and remove non-peptide peaks prior to database searches performed by the Mascot search engine [22].

## Results and discussion

### A simple way to predict the peptide mass cluster centres of a protein database

**Figure 1:**
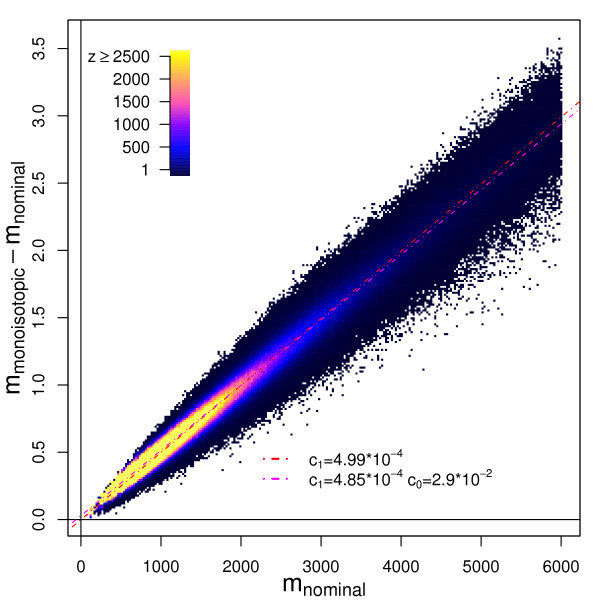
The peptide mass rule. Panel A: Scatterplot of *m*^(*M*) ^- *m*^(*N*) ^against the *m*^(*N*) ^mass (*m*^(*M*) ^- monoisotopic mass, *m*^(*N*) ^- *nominalmass)*. Red dashed line – the model determined by linear regression with intercept fixed at 0. The magenta line represents the cluster centres predicted by linear regression.

Figure 1 shows the mass defect, the difference of the monoisotopic (*m*^(*M*)^) and nominal (*m*^(*N*)^)masses of peptides of a sequence specific *in silico *protein sequence database digest [27], as a function of *m*^(*N*)^. The peptides were produced with the restriction that no missed cleavages were allowed. A strong linear dependence of the mass defect on *m*^(*N*) ^can be observed.

The first model of this dependence which we examined was *m*^(*M*) ^- *m*^(*N*) ^= *c*_1_·*m*^(*N*)^. We fixed the intercept at 0, because a hypothetical peptide with a nominal mass of 0 must have a monoisotopic mass equal to 0. The slope coefficient *c*_1_, determined by linear regression (cf. Methods) equalled 4.98·10^-4^(Figure 1, Panel A – red dashed line), which is a value similar to the values 4.95·10^-4 ^reported by Wool and Smilansky [10].

We were interested in determining the dependence between monoisotopic and nominal mass analytically.

For example, the monoisotopic mass (*m*^(*M*)^) of hypothetical peptides built only of one amino acid *i *can be predicted, given their nominal mass (*m*^(*N*)^) by mi(M)
 MathType@MTEF@5@5@+=feaafiart1ev1aaatCvAUfKttLearuWrP9MDH5MBPbIqV92AaeXatLxBI9gBaebbnrfifHhDYfgasaacH8akY=wiFfYdH8Gipec8Eeeu0xXdbba9frFj0=OqFfea0dXdd9vqai=hGuQ8kuc9pgc9s8qqaq=dirpe0xb9q8qiLsFr0=vr0=vr0dc8meaabaqaciaacaGaaeqabaqabeGadaaakeaacqWGTbqBdaqhaaWcbaGaemyAaKgabaWaaeWaceaacqWGnbqtaiaawIcacaGLPaaaaaaaaa@3245@ = *λ*_*i*_mi(N)
 MathType@MTEF@5@5@+=feaafiart1ev1aaatCvAUfKttLearuWrP9MDH5MBPbIqV92AaeXatLxBI9gBaebbnrfifHhDYfgasaacH8akY=wiFfYdH8Gipec8Eeeu0xXdbba9frFj0=OqFfea0dXdd9vqai=hGuQ8kuc9pgc9s8qqaq=dirpe0xb9q8qiLsFr0=vr0=vr0dc8meaabaqaciaacaGaaeqabaqabeGadaaakeaacqWGTbqBdaqhaaWcbaGaemyAaKgabaWaaeWaceaacqWGobGtaiaawIcacaGLPaaaaaaaaa@3247@ when *λ*_*i *_= mi(M)
 MathType@MTEF@5@5@+=feaafiart1ev1aaatCvAUfKttLearuWrP9MDH5MBPbIqV92AaeXatLxBI9gBaebbnrfifHhDYfgasaacH8akY=wiFfYdH8Gipec8Eeeu0xXdbba9frFj0=OqFfea0dXdd9vqai=hGuQ8kuc9pgc9s8qqaq=dirpe0xb9q8qiLsFr0=vr0=vr0dc8meaabaqaciaacaGaaeqabaqabeGadaaakeaacqWGTbqBdaqhaaWcbaGaemyAaKgabaWaaeWaceaacqWGnbqtaiaawIcacaGLPaaaaaaaaa@3245@/mi(N)
 MathType@MTEF@5@5@+=feaafiart1ev1aaatCvAUfKttLearuWrP9MDH5MBPbIqV92AaeXatLxBI9gBaebbnrfifHhDYfgasaacH8akY=wiFfYdH8Gipec8Eeeu0xXdbba9frFj0=OqFfea0dXdd9vqai=hGuQ8kuc9pgc9s8qqaq=dirpe0xb9q8qiLsFr0=vr0=vr0dc8meaabaqaciaacaGaaeqabaqabeGadaaakeaacqWGTbqBdaqhaaWcbaGaemyAaKgabaWaaeWaceaacqWGobGtaiaawIcacaGLPaaaaaaaaa@3247@. For peptides generated by random cleavage of protein sequences from a protein database this dependence is approximated by:

λDB=∑i∈AAfimi(M)∑i∈AAfimi(N),     (2)
 MathType@MTEF@5@5@+=feaafiart1ev1aaatCvAUfKttLearuWrP9MDH5MBPbIqV92AaeXatLxBI9gBaebbnrfifHhDYfgasaacH8akY=wiFfYdH8Gipec8Eeeu0xXdbba9frFj0=OqFfea0dXdd9vqai=hGuQ8kuc9pgc9s8qqaq=dirpe0xb9q8qiLsFr0=vr0=vr0dc8meaabaqaciaacaGaaeqabaqabeGadaaakeaaiiGacqWF7oaBdaWgaaWcbaGaemiraqKaemOqaieabeaakiabg2da9maalaaabaWaaabeaeaacqWGMbGzdaWgaaWcbaGaemyAaKgabeaakiabd2gaTnaaDaaaleaacqWGPbqAaeaadaqadiqaaiabd2eanbGaayjkaiaawMcaaaaaaeaacqWGPbqAcqGHiiIZcqWGbbqqcqWGbbqqaeqaniabggHiLdaakeaadaaeqaqaaiabdAgaMnaaBaaaleaacqWGPbqAaeqaaOGaemyBa02aa0baaSqaaiabdMgaPbqaamaabmGabaGaemOta4eacaGLOaGaayzkaaaaaaqaaiabdMgaPjabgIGiolabdgeabjabdgeabbqab0GaeyyeIuoaaaGccqGGSaalcaWLjaGaaCzcamaabmGabaGaeGOmaidacaGLOaGaayzkaaaaaa@5520@

where *f*_*i *_is the frequency of the amino acid *i *in the database.

Now write mi(M)
 MathType@MTEF@5@5@+=feaafiart1ev1aaatCvAUfKttLearuWrP9MDH5MBPbIqV92AaeXatLxBI9gBaebbnrfifHhDYfgasaacH8akY=wiFfYdH8Gipec8Eeeu0xXdbba9frFj0=OqFfea0dXdd9vqai=hGuQ8kuc9pgc9s8qqaq=dirpe0xb9q8qiLsFr0=vr0=vr0dc8meaabaqaciaacaGaaeqabaqabeGadaaakeaacqWGTbqBdaqhaaWcbaGaemyAaKgabaWaaeWaceaacqWGnbqtaiaawIcacaGLPaaaaaaaaa@3245@ = *λ*_*DB*_mi(N)
 MathType@MTEF@5@5@+=feaafiart1ev1aaatCvAUfKttLearuWrP9MDH5MBPbIqV92AaeXatLxBI9gBaebbnrfifHhDYfgasaacH8akY=wiFfYdH8Gipec8Eeeu0xXdbba9frFj0=OqFfea0dXdd9vqai=hGuQ8kuc9pgc9s8qqaq=dirpe0xb9q8qiLsFr0=vr0=vr0dc8meaabaqaciaacaGaaeqabaqabeGadaaakeaacqWGTbqBdaqhaaWcbaGaemyAaKgabaWaaeWaceaacqWGobGtaiaawIcacaGLPaaaaaaaaa@3247@ + *ε*_*i*_. Substituting this is (2), it follows that ∑_*i*∈*AA *_*f*_*i*_*ε*_*i *_= 0. Therefore, for an amino acid randomly selected from the database, with frequencies *f*_*i*_, the expectation of *ε*_*i *_is zero. Now consider a peptide made of a random selection of *J *amino acids, *i*(1),...,*i*(*J*). The ratio of monoisotopic to nominal mass for this peptide would be:

λp=∑j=1Jmi(j)M∑j=1Jmi(j)N=λDB∑j=1Jmi(j)N+∑j=1Jεi(j)∑j=1Jmi(j)N.
 MathType@MTEF@5@5@+=feaafiart1ev1aaatCvAUfKttLearuWrP9MDH5MBPbIqV92AaeXatLxBI9gBaebbnrfifHhDYfgasaacH8akY=wiFfYdH8Gipec8Eeeu0xXdbba9frFj0=OqFfea0dXdd9vqai=hGuQ8kuc9pgc9s8qqaq=dirpe0xb9q8qiLsFr0=vr0=vr0dc8meaabaqaciaacaGaaeqabaqabeGadaaakeaaiiGacqWF7oaBdaWgaaWcbaGaemiCaahabeaakiabg2da9maalaaabaWaaabmaeaacqWGTbqBdaqhaaWcbaGaemyAaK2aaeWaceaacqWGQbGAaiaawIcacaGLPaaaaeaacqWGnbqtaaaabaGaemOAaOMaeyypa0JaeGymaedabaGaemOsaOeaniabggHiLdaakeaadaaeWaqaaiabd2gaTnaaDaaaleaacqWGPbqAdaqadiqaaiabdQgaQbGaayjkaiaawMcaaaqaaiabd6eaobaaaeaacqWGQbGAcqGH9aqpcqaIXaqmaeaacqWGkbGsa0GaeyyeIuoaaaGccqGH9aqpdaWcaaqaaiab=T7aSnaaBaaaleaacqWGebarcqWGcbGqaeqaaOWaaabmaeaacqWGTbqBdaqhaaWcbaGaemyAaK2aaeWaceaacqWGQbGAaiaawIcacaGLPaaaaeaacqWGobGtaaaabaGaemOAaOMaeyypa0JaeGymaedabaGaemOsaOeaniabggHiLdGccqGHRaWkdaaeWaqaaiab=v7aLnaaBaaaleaacqWGPbqAdaqadiqaaiabdQgaQbGaayjkaiaawMcaaaqabaaabaGaemOAaOMaeyypa0JaeGymaedabaGaemOsaOeaniabggHiLdaakeaadaaeWaqaaiabd2gaTnaaDaaaleaacqWGPbqAdaqadiqaaiabdQgaQbGaayjkaiaawMcaaaqaaiabd6eaobaaaeaacqWGQbGAcqGH9aqpcqaIXaqmaeaacqWGkbGsa0GaeyyeIuoaaaGccqGGUaGlaaa@7A1F@

If ∑_*i *_*ε*_*i*(*j*) _were uncorrelated with (∑imi(j)(N))−1
 MathType@MTEF@5@5@+=feaafiart1ev1aaatCvAUfKttLearuWrP9MDH5MBPbIqV92AaeXatLxBI9gBaebbnrfifHhDYfgasaacH8akY=wiFfYdH8Gipec8Eeeu0xXdbba9frFj0=OqFfea0dXdd9vqai=hGuQ8kuc9pgc9s8qqaq=dirpe0xb9q8qiLsFr0=vr0=vr0dc8meaabaqaciaacaGaaeqabaqabeGadaaakeaadaqadiqaamaaqababaGaemyBa02aa0baaSqaaiabdMgaPnaabmGabaGaemOAaOgacaGLOaGaayzkaaaabaWaaeWaceaacqWGobGtaiaawIcacaGLPaaaaaaabaGaemyAaKgabeqdcqGHris5aaGccaGLOaGaayzkaaWaaWbaaSqabeaacqGHsislcqaIXaqmaaaaaa@3C01@ for a random selection of amino acids, then *λ*_*p *_would have expectation *λ*_*DB*_. Of course, there may be a relationship between *ε*_*i *_and mi(N)
 MathType@MTEF@5@5@+=feaafiart1ev1aaatCvAUfKttLearuWrP9MDH5MBPbIqV92AaeXatLxBI9gBaebbnrfifHhDYfgasaacH8akY=wiFfYdH8Gipec8Eeeu0xXdbba9frFj0=OqFfea0dXdd9vqai=hGuQ8kuc9pgc9s8qqaq=dirpe0xb9q8qiLsFr0=vr0=vr0dc8meaabaqaciaacaGaaeqabaqabeGadaaakeaacqWGTbqBdaqhaaWcbaGaemyAaKgabaWaaeWaceaacqWGobGtaiaawIcacaGLPaaaaaaaaa@3247@ and we would wish to use any such relationship to improve prediction of mi(M)
 MathType@MTEF@5@5@+=feaafiart1ev1aaatCvAUfKttLearuWrP9MDH5MBPbIqV92AaeXatLxBI9gBaebbnrfifHhDYfgasaacH8akY=wiFfYdH8Gipec8Eeeu0xXdbba9frFj0=OqFfea0dXdd9vqai=hGuQ8kuc9pgc9s8qqaq=dirpe0xb9q8qiLsFr0=vr0=vr0dc8meaabaqaciaacaGaaeqabaqabeGadaaakeaacqWGTbqBdaqhaaWcbaGaemyAaKgabaWaaeWaceaacqWGnbqtaiaawIcacaGLPaaaaaaaaa@3245@

**Figure 2:**
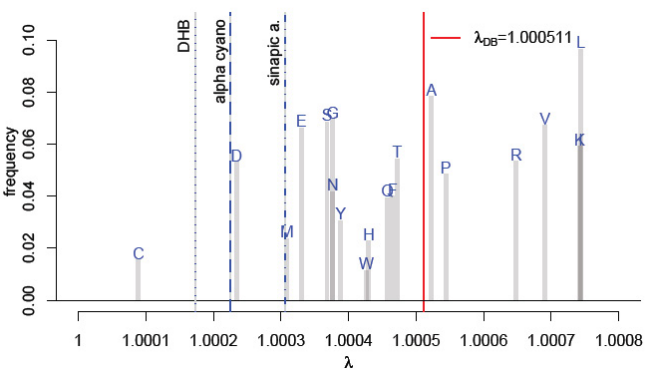
Bar-plot of the Amino Acid frequencies. The bars are drawn on the position of *λ*_*i *_= mi(M)
 MathType@MTEF@5@5@+=feaafiart1ev1aaatCvAUfKttLearuWrP9MDH5MBPbIqV92AaeXatLxBI9gBaebbnrfifHhDYfgasaacH8akY=wiFfYdH8Gipec8Eeeu0xXdbba9frFj0=OqFfea0dXdd9vqai=hGuQ8kuc9pgc9s8qqaq=dirpe0xb9q8qiLsFr0=vr0=vr0dc8meaabaqaciaacaGaaeqabaqabeGadaaakeaacqWGTbqBdaqhaaWcbaGaemyAaKgabaWaaeWaceaacqWGnbqtaiaawIcacaGLPaaaaaaaaa@3245@/mi(N)
 MathType@MTEF@5@5@+=feaafiart1ev1aaatCvAUfKttLearuWrP9MDH5MBPbIqV92AaeXatLxBI9gBaebbnrfifHhDYfgasaacH8akY=wiFfYdH8Gipec8Eeeu0xXdbba9frFj0=OqFfea0dXdd9vqai=hGuQ8kuc9pgc9s8qqaq=dirpe0xb9q8qiLsFr0=vr0=vr0dc8meaabaqaciaacaGaaeqabaqabeGadaaakeaacqWGTbqBdaqhaaWcbaGaemyAaKgabaWaaeWaceaacqWGobGtaiaawIcacaGLPaaaaaaaaa@3247@, for each amino acid *i*. The red line indicates *λ*_*DB *_computed using the Equation 2. Dotted blue line - *λ*_*DHB *_2,5-dihydroxybenzoic acid; dashed line - *λ*_*alphacyano *_alpha-Cyano-4-hydroxycinnamic acid; dot dashed line - *λ*_*sinapica*_. 3,5-Dimethoxy-4-hydroxycinnamic acid.

Figure 2 visualises the frequencies *f*_*i *_of all amino acids in the *Uniprot *database [27] with their respective *λ*_*i *_plotted on the abscissa. The position of the red vertical line on the abscissa denotes *λ*_*DB *_(Equation 2) and equals *λ*_*DB *_= 1.000511. The dotted, dashed and dot dashed lines indicate the wavelength *λ *of DHB, alpha-cyano and sinapic acid mass spectrometric matrix clusters, respectively.

When testing for the significance of the intercept coefficient in the regression model *m*_*M *_∝ *λm*_*N *_of a sequence specific (Tryptic) *in silico *database digest, we found that the intercept coefficient must be included into the model. Therefore, the extended model of the monoisotopic peptide mass cluster centres was:

*m*^(*M*) ^= *c*_1_·*m*^(*N*) ^+ *c*_0_.     (3)

Subtracting *m*_*N *_from each side of Equation 3 we obtained Δ = *m*^(*M*) ^- *m*^(*N*) ^= (*c*_1 _- 1)·*m*^(*N*) ^+ *c*_0_. The coefficients of the affine linear model of the cluster centres, determined using regression analysis of Δ = *m*^(*M*) ^- *m*^(*N*) ^on *m*^(*N*) ^were *c*_0 _= 0.029 and (*c*_1 _- 1) = 4.85·10^-4^.

The maximal difference between the prediction of *m*^(*M*) ^using *m*^(*M*) ^= 1.000499·*m*^(*N*) ^and *m*^(*M*) ^= 1.000485·*m*^(*N*) ^+ 0.029 is 0.022 Dalton for *m*^(*N*) ^∈ [600, 2500] Dalton.

### The influence of the digestion enzyme on the wavelength of peptide mass clusters

In case of a complete sequence specific cleavage of proteins, the number of generated peptides is *C*_*P *_+ 1 peptides, given that *C*_*P *_is the number of cleavage sites per protein. The peptides generated from the terminus of the protein (further called *terminal*) will not bear a cleavage site residue *R*_*C *_at their end. All the other peptides, which we call *internal*, will have such a residue at their end. The fraction of the internal peptides *f*_*c*,*n *_is given by

fc,n=CP−nCP+1−n,     (4)
 MathType@MTEF@5@5@+=feaafiart1ev1aaatCvAUfKttLearuWrP9MDH5MBPbIqV92AaeXatLxBI9gBaebbnrfifHhDYfgasaacH8akY=wiFfYdH8Gipec8Eeeu0xXdbba9frFj0=OqFfea0dXdd9vqai=hGuQ8kuc9pgc9s8qqaq=dirpe0xb9q8qiLsFr0=vr0=vr0dc8meaabaqaciaacaGaaeqabaqabeGadaaakeaacqWGMbGzdaWgaaWcbaGaem4yamMaeiilaWIaemOBa4gabeaakiabg2da9maalaaabaGaem4qam0aaSbaaSqaaiabdcfaqbqabaGccqGHsislcqWGUbGBaeaacqWGdbWqdaWgaaWcbaGaemiuaafabeaakiabgUcaRiabigdaXiabgkHiTiabd6gaUbaacqGGSaalcaWLjaGaaCzcamaabmGabaGaeGinaqdacaGLOaGaayzkaaaaaa@42D8@

where *n *is the number of missed cleavages per protein. We approximate *C*_*P*_, for a sequence database, by:

CP=|P|⋅(∑fRC),     (5)
 MathType@MTEF@5@5@+=feaafiart1ev1aaatCvAUfKttLearuWrP9MDH5MBPbIqV92AaeXatLxBI9gBaebbnrfifHhDYfgasaacH8akY=wiFfYdH8Gipec8Eeeu0xXdbba9frFj0=OqFfea0dXdd9vqai=hGuQ8kuc9pgc9s8qqaq=dirpe0xb9q8qiLsFr0=vr0=vr0dc8meaabaqaciaacaGaaeqabaqabeGadaaakeaacqWGdbWqdaWgaaWcbaGaemiuaafabeaakiabg2da9iabcYha8jabdcfaqjabcYha8jabgwSixpaabmGabaWaaabqaeaacqWGMbGzdaWgaaWcbaGaemOuai1aaSbaaWqaaiabdoeadbqabaaaleqaaaqabeqaniabggHiLdaakiaawIcacaGLPaaacqGGSaalcaWLjaGaaCzcamaabmGabaGaeGynaudacaGLOaGaayzkaaaaaa@42CD@

where fRC
 MathType@MTEF@5@5@+=feaafiart1ev1aaatCvAUfKttLearuWrP9MDH5MBPbIqV92AaeXatLxBI9gBaebbnrfifHhDYfgasaacH8akY=wiFfYdH8Gipec8Eeeu0xXdbba9frFj0=OqFfea0dXdd9vqai=hGuQ8kuc9pgc9s8qqaq=dirpe0xb9q8qiLsFr0=vr0=vr0dc8meaabaqaciaacaGaaeqabaqabeGadaaakeaacqWGMbGzdaWgaaWcbaGaemOuai1aaSbaaWqaaiabdoeadbqabaaaleqaaaaa@30A1@ are the relative frequencies of the cleavage sites and |*P*| is the average protein length in the database. The fraction of the terminal peptides in case of *n *missed cleavages is given by 1 - *f*_*c*,*n*_. The fraction of cleavage site residues *R*_*C *_in a internal peptide of mass *m*_pep_, with *n *missed cleavage sites is denoted *f*_*m*,*n *_and approximated by:

fm,n=(n+1)m¯mpep,     (6)
 MathType@MTEF@5@5@+=feaafiart1ev1aaatCvAUfKttLearuWrP9MDH5MBPbIqV92AaeXatLxBI9gBaebbnrfifHhDYfgasaacH8akY=wiFfYdH8Gipec8Eeeu0xXdbba9frFj0=OqFfea0dXdd9vqai=hGuQ8kuc9pgc9s8qqaq=dirpe0xb9q8qiLsFr0=vr0=vr0dc8meaabaqaciaacaGaaeqabaqabeGadaaakeaacqWGMbGzdaWgaaWcbaGaemyBa0MaeiilaWIaemOBa4gabeaakiabg2da9maabmGabaGaemOBa4Maey4kaSIaeGymaedacaGLOaGaayzkaaWaaSaaaeaacuWGTbqBgaqeaaqaaiabd2gaTnaaBaaaleaacqqGWbaCcqqGLbqzcqqGWbaCaeqaaaaakiabcYcaSiaaxMaacaWLjaWaaeWaceaacqaI2aGnaiaawIcacaGLPaaaaaa@4393@

where m¯
 MathType@MTEF@5@5@+=feaafiart1ev1aaatCvAUfKttLearuWrP9MDH5MBPbIqV92AaeXatLxBI9gBaebbnrfifHhDYfgasaacH8akY=wiFfYdH8Gipec8Eeeu0xXdbba9frFj0=OqFfea0dXdd9vqai=hGuQ8kuc9pgc9s8qqaq=dirpe0xb9q8qiLsFr0=vr0=vr0dc8meaabaqaciaacaGaaeqabaqabeGadaaakeaacuWGTbqBgaqeaaaa@2E27@ is the average mass of an amino acid residue. A more accurate model of *f*_*m*,*n *_is provided in the *Appendix*. In the case of terminal peptides the fraction of cleavage site residues *R*_*C *_equals *f*_*m*,*n *- 1_. The fraction of all the other amino acid residues *R*\*R*_*C *_equals 1 - *f*_*m*,*n *_or 1 - *f*_*m*,*n *- 1 _respectively. Table 2 summarises these results.

**Table 2:** Frequencies of cleavage site residues, and all other residues, in peptides of mass *m *and of terminal, and internal, peptides.

*R*_non-cleavage_	*R*_cleavage_		Peptide type
(1 - *f*_*m*,*n*_)	*f*_*m*,*n*_	*f*_*c*,*n*_	internal
(1 - *f*_*m*,*n *- 1_)	*f*_*m*,*n *- 1_	1 - *f*_*c*,*n*_	terminal

*R*_cleavage _– frequencies of cleavage site residues; *R*_non-cleavage _– frequencies of non-cleavage site residues; *f*_*m*,*n *_– see Equation 6; *f*_*c*,*n *_– see Equation 4.

In the case of internal peptides, the average contribution of the amino acid residues to the peptide mass is the weighted sum:

mRC,n(∗)=(1−fm,n)⋅mnone+fm,n⋅mRc     (7)=mnone+fm,n⋅(mRC−mnone),(8)
 MathType@MTEF@5@5@+=feaafiart1ev1aaatCvAUfKttLearuWrP9MDH5MBPbIqV92AaeXatLxBI9gBaebbnrfifHhDYfgasaacH8akY=wiFfYdH8Gipec8Eeeu0xXdbba9frFj0=OqFfea0dXdd9vqai=hGuQ8kuc9pgc9s8qqaq=dirpe0xb9q8qiLsFr0=vr0=vr0dc8meaabaqaciaacaGaaeqabaqabeGadaaakeaafaqadeGacaaabaGaemyBa02aa0baaSqaaiabdkfasnaaBaaameaacqWGdbWqaeqaaSGaeiilaWIaemOBa4gabaWaaeWaceaacqGHxiIkaiaawIcacaGLPaaaaaGccqGH9aqpdaqadiqaaiabigdaXiabgkHiTiabdAgaMnaaBaaaleaacqWGTbqBcqGGSaalcqWGUbGBaeqaaaGccaGLOaGaayzkaaGaeyyXICTaemyBa02aaSbaaSqaaiabd6gaUjabd+gaVjabd6gaUjabdwgaLbqabaGccqGHRaWkcqWGMbGzdaWgaaWcbaGaemyBa0MaeiilaWIaemOBa4gabeaakiabgwSixlabd2gaTnaaBaaaleaacqWGsbGudaWgaaadbaGaem4yamgabeaaaSqabaGccaWLjaGaaCzcaaqaamaabmGabaGaeG4naCdacaGLOaGaayzkaaaabaGaeyypa0JaemyBa02aaSbaaSqaaiabd6gaUjabd+gaVjabd6gaUjabdwgaLbqabaGccqGHRaWkcqWGMbGzdaWgaaWcbaGaemyBa0MaeiilaWIaemOBa4gabeaakiabgwSixpaabmGabaGaemyBa02aaSbaaSqaaiabdkfasnaaBaaameaacqWGdbWqaeqaaaWcbeaakiabgkHiTiabd2gaTnaaBaaaleaacqWGUbGBcqWGVbWBcqWGUbGBcqWGLbqzaeqaaaGccaGLOaGaayzkaaGaeiilaWcabaWaaeWaceaacqaI4aaoaiaawIcacaGLPaaaaaaaaa@7A97@

where

mnone=∑i∈R\RCfi⋅mi,     (9)
 MathType@MTEF@5@5@+=feaafiart1ev1aaatCvAUfKttLearuWrP9MDH5MBPbIqV92AaeXatLxBI9gBaebbnrfifHhDYfgasaacH8akY=wiFfYdH8Gipec8Eeeu0xXdbba9frFj0=OqFfea0dXdd9vqai=hGuQ8kuc9pgc9s8qqaq=dirpe0xb9q8qiLsFr0=vr0=vr0dc8meaabaqaciaacaGaaeqabaqabeGadaaakeaacqWGTbqBdaWgaaWcbaGaemOBa4Maem4Ba8MaemOBa4Maemyzaugabeaakiabg2da9maaqafabaGaemOzay2aaSbaaSqaaiabdMgaPbqabaGccqGHflY1cqWGTbqBdaWgaaWcbaGaemyAaKgabeaakiabcYcaSaWcbaGaemyAaKMaeyicI4SaemOuaiLaeiixaWLaemOuai1aaSbaaWqaaiabdoeadbqabaaaleqaniabggHiLdGccaWLjaGaaCzcamaabmGabaGaeGyoaKdacaGLOaGaayzkaaaaaa@4B8F@

is the average mass of non cleavage residues, and:

mRC=∑i∈RCfi⋅mi⋅     (10)
 MathType@MTEF@5@5@+=feaafiart1ev1aaatCvAUfKttLearuWrP9MDH5MBPbIqV92AaeXatLxBI9gBaebbnrfifHhDYfgasaacH8akY=wiFfYdH8Gipec8Eeeu0xXdbba9frFj0=OqFfea0dXdd9vqai=hGuQ8kuc9pgc9s8qqaq=dirpe0xb9q8qiLsFr0=vr0=vr0dc8meaabaqaciaacaGaaeqabaqabeGadaaakeaacqWGTbqBdaWgaaWcbaGaemOuai1aaSbaaWqaaiabdoeadbqabaaaleqaaOGaeyypa0ZaaabuaeaacqWGMbGzdaWgaaWcbaGaemyAaKgabeaakiabgwSixlabd2gaTnaaBaaaleaacqWGPbqAcqGHflY1aeqaaaqaaiabdMgaPjabgIGiolabdkfasnaaBaaameaacqWGdbWqaeqaaaWcbeqdcqGHris5aOGaaCzcaiaaxMaadaqadiqaaiabigdaXiabicdaWaGaayjkaiaawMcaaaaa@4845@

is the average mass of the cleavage site residues *R*_*C*_. Finally, the wavelength of internal peptides is presented as:

λRC,nm=mRC,n(M)mRC,n(N)     (11)
 MathType@MTEF@5@5@+=feaafiart1ev1aaatCvAUfKttLearuWrP9MDH5MBPbIqV92AaeXatLxBI9gBaebbnrfifHhDYfgasaacH8akY=wiFfYdH8Gipec8Eeeu0xXdbba9frFj0=OqFfea0dXdd9vqai=hGuQ8kuc9pgc9s8qqaq=dirpe0xb9q8qiLsFr0=vr0=vr0dc8meaabaqaciaacaGaaeqabaqabeGadaaakeaaiiGacqWF7oaBdaqhaaWcbaGaemOuai1aaSbaaWqaaiabdoeadbqabaWccqGGSaalcqWGUbGBaeaacqWGTbqBaaGccqGH9aqpdaWcaaqaaiabd2gaTnaaDaaaleaacqWGsbGudaWgaaadbaGaem4qameabeaaliabcYcaSiabd6gaUbqaamaabmGabaGaemyta0eacaGLOaGaayzkaaaaaaGcbaGaemyBa02aa0baaSqaaiabdkfasnaaBaaameaacqWGdbWqaeqaaSGaeiilaWIaemOBa4gabaWaaeWaceaacqWGobGtaiaawIcacaGLPaaaaaaaaOGaaCzcaiaaxMaadaqadiqaaiabigdaXiabigdaXaGaayjkaiaawMcaaaaa@4C83@

The wavelength of terminal peptides was determined by: λRC,m(n−1)=mRC,n−1(M)mRC,n−1(N)
 MathType@MTEF@5@5@+=feaafiart1ev1aaatCvAUfKttLearuWrP9MDH5MBPbIqV92AaeXatLxBI9gBaebbnrfifHhDYfgasaacH8akY=wiFfYdH8Gipec8Eeeu0xXdbba9frFj0=OqFfea0dXdd9vqai=hGuQ8kuc9pgc9s8qqaq=dirpe0xb9q8qiLsFr0=vr0=vr0dc8meaabaqaciaacaGaaeqabaqabeGadaaakeaaiiGacqWF7oaBdaqhaaWcbaGaemOuai1aaSbaaWqaaiabdoeadbqabaWccqGGSaalcqWGTbqBaeaadaqadiqaaiabd6gaUjabgkHiTiabigdaXaGaayjkaiaawMcaaaaakiabg2da9maalaaabaGaemyBa02aa0baaSqaaiabdkfasnaaBaaameaacqWGdbWqaeqaaSGaeiilaWIaemOBa4MaeyOeI0IaeGymaedabaWaaeWaceaacqWGnbqtaiaawIcacaGLPaaaaaaakeaacqWGTbqBdaqhaaWcbaGaemOuai1aaSbaaWqaaiabdoeadbqabaWccqGGSaalcqWGUbGBcqGHsislcqaIXaqmaeaadaqadiqaaiabd6eaobGaayjkaiaawMcaaaaaaaaaaa@4EEC@.

**The wavelength ***λ ***of all peptides at a mass ***m ***with exactly ***n ***missed cleavages **is given by:

λRC,nm,∗=mRC,n(M),∗mRC,n(N),∗     (12)
 MathType@MTEF@5@5@+=feaafiart1ev1aaatCvAUfKttLearuWrP9MDH5MBPbIqV92AaeXatLxBI9gBaebbnrfifHhDYfgasaacH8akY=wiFfYdH8Gipec8Eeeu0xXdbba9frFj0=OqFfea0dXdd9vqai=hGuQ8kuc9pgc9s8qqaq=dirpe0xb9q8qiLsFr0=vr0=vr0dc8meaabaqaciaacaGaaeqabaqabeGadaaakeaaiiGacqWF7oaBdaqhaaWcbaGaemOuai1aaSbaaWqaaiabdoeadbqabaWccqGGSaalcqWGUbGBaeaacqWGTbqBcqGGSaalcqGHxiIkaaGccqGH9aqpdaWcaaqaaiabd2gaTnaaDaaaleaacqWGsbGudaWgaaadbaGaem4qameabeaaliabcYcaSiabd6gaUbqaamaabmGabaGaemyta0eacaGLOaGaayzkaaGaeiilaWIaey4fIOcaaaGcbaGaemyBa02aa0baaSqaaiabdkfasnaaBaaameaacqWGdbWqaeqaaSGaeiilaWIaemOBa4gabaWaaeWaceaacqWGobGtaiaawIcacaGLPaaacqGGSaalcqGHxiIkaaaaaOGaaCzcaiaaxMaadaqadiqaaiabigdaXiabikdaYaGaayjkaiaawMcaaaaa@51F2@

where

mRC,n[MN],∗=fc,n⋅mRC,n[MN]+(1−fc,n)⋅mRC,n−1[MN](13)=mnone+(mRC−mnone)⋅(fc,nfm,n+fm,(n−1)−fc,nfm,(n−1))     (14)=︸with Equation 6mnone+m¯m(fc,n+n)(mRC−mnone)(15)=︸with Equation 4mnone+(Cp−nCp+1−n+n)⋅m¯m⋅(mRC−mnone)(16)
 MathType@MTEF@5@5@+=feaafiart1ev1aaatCvAUfKttLearuWrP9MDH5MBPbIqV92AaeXatLxBI9gBaebbnrfifHhDYfgasaacH8akY=wiFfYdH8Gipec8Eeeu0xXdbba9frFj0=OqFfea0dXdd9vqai=hGuQ8kuc9pgc9s8qqaq=dirpe0xb9q8qiLsFr0=vr0=vr0dc8meaabaqaciaacaGaaeqabaqabeGadaaakeaafaqaaeabeaaaaaqaaiabd2gaTnaaDaaaleaacqWGsbGudaWgaaadbaGaem4qameabeaaliabcYcaSiabd6gaUbqaaiabcUfaBjabd2eanjabd6eaojabc2faDjabcYcaSiabgEHiQaaaaOqaceaaDlGaaCzcaiabg2da9aqaaiabdAgaMnaaBaaaleaacqWGJbWycqGGSaalcqWGUbGBaeqaaOGaeyyXICTaemyBa02aa0baaSqaaiabdkfasnaaBaaameaacqWGdbWqaeqaaSGaeiilaWIaemOBa4gabaGaei4waSLaemyta0KaemOta4Kaeiyxa0faaOGaey4kaSIaeiikaGIaeGymaeJaeyOeI0IaemOzay2aaSbaaSqaaiabdogaJjabcYcaSiabd6gaUbqabaGccqGGPaqkcqGHflY1cqWGTbqBdaqhaaWcbaGaemOuai1aaSbaaWqaaiabdoeadbqabaWccqGGSaalcqWGUbGBcqGHsislcqaIXaqmaeaacqGGBbWwcqWGnbqtcqWGobGtcqGGDbqxaaaakeaadaqadiqaaiabigdaXiabiodaZaGaayjkaiaawMcaaaqaaaqaceaaPlGaaCzcaiabg2da9aqaaiabd2gaTnaaBaaaleaacqWGUbGBcqWGVbWBcqWGUbGBcqWGLbqzaeqaaOGaey4kaSYaaeWaceaacqWGTbqBdaWgaaWcbaGaemOuai1aaSbaaWqaaiabdoeadbqabaaaleqaaOGaeyOeI0IaemyBa02aaSbaaSqaaiabd6gaUjabd+gaVjabd6gaUjabdwgaLbqabaaakiaawIcacaGLPaaacqGHflY1daqadiqaaiabdAgaMnaaBaaaleaacqWGJbWycqGGSaalcqWGUbGBaeqaaOGaemOzay2aaSbaaSqaaiabd2gaTjabcYcaSiabd6gaUbqabaGccqGHRaWkcqWGMbGzdaWgaaWcbaGaemyBa0MaeiilaWIaeiikaGIaemOBa4MaeyOeI0IaeGymaeJaeiykaKcabeaakiabgkHiTiabdAgaMnaaBaaaleaacqWGJbWycqGGSaalcqWGUbGBaeqaaOGaemOzay2aaSbaaSqaaiabd2gaTjabcYcaSiabcIcaOiabd6gaUjabgkHiTiabigdaXiabcMcaPaqabaaakiaawIcacaGLPaaacaWLjaGaaCzcaaqaamaabmGabaGaeGymaeJaeGinaqdacaGLOaGaayzkaaaabaaabaWaaGbaaeaacqGH9aqpaSqaaiabbEha3jabbMgaPjabbsha0jabbIgaOjabbccaGiabbweafjabbghaXjabbwha1jabbggaHjabbsha0jabbMgaPjabb+gaVjabb6gaUjabbccaGiabbAda2aGccaGL44paaeaacqWGTbqBdaWgaaWcbaGaemOBa4Maem4Ba8MaemOBa4MaemyzaugabeaakiabgUcaRmaalaaabaGafmyBa0MbaebaaeaacqWGTbqBaaWaaeWaceaacqWGMbGzdaWgaaWcbaGaem4yamMaeiilaWIaemOBa4gabeaakiabgUcaRiabd6gaUbGaayjkaiaawMcaamaabmGabaGaemyBa02aaSbaaSqaaiabdkfasnaaBaaameaacqWGdbWqaeqaaaWcbeaakiabgkHiTiabd2gaTnaaBaaaleaacqWGUbGBcqWGVbWBcqWGUbGBcqWGLbqzaeqaaaGccaGLOaGaayzkaaaabaWaaeWaceaacqaIXaqmcqaI1aqnaiaawIcacaGLPaaaaeaaaeaadaagaaqaaiabg2da9aWcbaGaee4DaCNaeeyAaKMaeeiDaqNaeeiAaGMaeeiiaaIaeeyrauKaeeyCaeNaeeyDauNaeeyyaeMaeeiDaqNaeeyAaKMaee4Ba8MaeeOBa4MaeeiiaaIaeeinaqdakiaawIJ=aaqaaGqadiab=1gaTnaaBaaaleaacqWFUbGBcqWFVbWBcqWFUbGBcqWFLbqzaeqaaOGaey4kaSYaaeWaceaadaWcaaqaaiab=neadnaaBaaaleaacqWFWbaCaeqaaOGaeyOeI0Iae8NBa4gabaGae83qam0aaSbaaSqaaiab=bhaWbqabaGccqGHRaWkieqacqGFXaqmcqGHsislcqWFUbGBaaGaey4kaSIae8NBa4gacaGLOaGaayzkaaGaeyyXIC9aaSaaaeaacuWFTbqBgaqeaaqaaiab=1gaTbaacqGHflY1daqadiqaaiab=1gaTnaaBaaaleaacqWFsbGudaWgaaadbaGae83qameabeaaaSqabaGccqGHsislcqWFTbqBdaWgaaWcbaGae8NBa4Mae83Ba8Mae8NBa4Mae8xzaugabeaaaOGaayjkaiaawMcaaaqaamaabmGabaGaeGymaeJaeGOnaydacaGLOaGaayzkaaaaaaaa@2F36@

is the weighted sum of the mass of the terminal peptides (with frequency 1 - *f*_*c*,*n*_) and the internal peptides (with frequency *f*_*c*,*n*_).

**Cleavage probability ***p*_*c *_In practice, the cleavage probability will depend on various factors, for example on the incubation time and the efficiency of the protease used. The probability to generate a peptide with *n *∈ 0...∞ missed cleavage sites, given the cleavage probability *p*_*c *_can be modelled using the geometric distribution:

*P*(*n*, *p*_*c*_) = (1 - *p*_*c*_)^*n*^·*p*_*c *_    (17)

Furthermore,

∑n=0∞(1−pc)n⋅pc=1     (18)
 MathType@MTEF@5@5@+=feaafiart1ev1aaatCvAUfKttLearuWrP9MDH5MBPbIqV92AaeXatLxBI9gBaebbnrfifHhDYfgasaacH8akY=wiFfYdH8Gipec8Eeeu0xXdbba9frFj0=OqFfea0dXdd9vqai=hGuQ8kuc9pgc9s8qqaq=dirpe0xb9q8qiLsFr0=vr0=vr0dc8meaabaqaciaacaGaaeqabaqabeGadaaakeaadaaeWbqaamaabmGabaGaeGymaeJaeyOeI0IaemiCaa3aaSbaaSqaaiabdogaJbqabaaakiaawIcacaGLPaaadaahaaWcbeqaaiabd6gaUbaaaeaacqWGUbGBcqGH9aqpcqaIWaamaeaacqGHEisPa0GaeyyeIuoakiabgwSixlabdchaWnaaBaaaleaacqWGJbWyaeqaaOGaeyypa0JaeGymaeJaaCzcaiaaxMaadaqadiqaaiabigdaXiabiIda4aGaayjkaiaawMcaaaaa@478A@

holds. Hence, given the cleavage probability is *p*_*c*_and cleavage residues *R*_*C*_, we express the peptide mass by:

mRC,pc∗=mnone+∑n=0∞(1−pc)n⋅pc⋅(mRC−mnone)⋅Sn,     (19)
 MathType@MTEF@5@5@+=feaafiart1ev1aaatCvAUfKttLearuWrP9MDH5MBPbIqV92AaeXatLxBI9gBaebbnrfifHhDYfgasaacH8akY=wiFfYdH8Gipec8Eeeu0xXdbba9frFj0=OqFfea0dXdd9vqai=hGuQ8kuc9pgc9s8qqaq=dirpe0xb9q8qiLsFr0=vr0=vr0dc8meaabaqaciaacaGaaeqabaqabeGadaaakeaacqWGTbqBdaqhaaWcbaGaemOuai1aaSbaaWqaaiabdoeadbqabaWccqGGSaalcqWGWbaCdaWgaaadbaGaem4yamgabeaaaSqaaiabgEHiQaaakiabg2da9Gqadiab=1gaTnaaBaaaleaacqWFUbGBcqWFVbWBcqWFUbGBcqWFLbqzaeqaaOGaey4kaSYaaabCaeaadaqadiqaaGqabiab+fdaXGGabiab9jHiTiab=bhaWnaaBaaaleaacqWFJbWyaeqaaaGccaGLOaGaayzkaaWaaWbaaSqabeaacqWFUbGBaaaabaGae8NBa4Mae0xpa0Jae4hmaadabaGae0NhIukaniabggHiLdGccqqFflY1cqWFWbaCdaWgaaWcbaGae83yamgabeaakiab9vSixpaabmGabaGae8xBa02aaSbaaSqaaiab=jfasnaaBaaameaacqWFdbWqaeqaaaWcbeaakiab9jHiTiab=1gaTnaaBaaaleaacqWFUbGBcqWFVbWBcqWFUbGBcqWFLbqzaeqaaaGccaGLOaGaayzkaaGae0xXICTae83uam1aaSbaaSqaaiab=5gaUbqabaGccqGGSaalcaWLjaGaaCzcamaabmGabaGaeGymaeJaeGyoaKdacaGLOaGaayzkaaaaaa@6CC2@

where

*S*_*n *_= (*f*_*c*,*n*_*f*_*m*,*n *_+ *f*_*m*,(*n*-1) _- *f*_*c*,*n*_*f*_*m*,(*n*-1)_).     (20)

Therefore, the wavelength *λ *of peptides if the cleavage probability is *p*_*c *_is given by:

λRC,pcm,∗=mRC,pc(M),∗mRC,pc(N),∗     (21)
 MathType@MTEF@5@5@+=feaafiart1ev1aaatCvAUfKttLearuWrP9MDH5MBPbIqV92AaeXatLxBI9gBaebbnrfifHhDYfgasaacH8akY=wiFfYdH8Gipec8Eeeu0xXdbba9frFj0=OqFfea0dXdd9vqai=hGuQ8kuc9pgc9s8qqaq=dirpe0xb9q8qiLsFr0=vr0=vr0dc8meaabaqaciaacaGaaeqabaqabeGadaaakeaaiiGacqWF7oaBdaqhaaWcbaGaemOuai1aaSbaaWqaaiabdoeadbqabaWccqGGSaalcqWGWbaCdaWgaaadbaGaem4yamgabeaaaSqaaiabd2gaTjabcYcaSiabgEHiQaaakiabg2da9maalaaabaGaemyBa02aa0baaSqaaiabdkfasnaaBaaameaacqWGdbWqaeqaaSGaeiilaWIaemiCaa3aaSbaaWqaaiabdogaJbqabaaaleaadaqadiqaaiabd2eanbGaayjkaiaawMcaaiabcYcaSiabgEHiQaaaaOqaaiabd2gaTnaaDaaaleaacqWGsbGudaWgaaadbaGaem4qameabeaaliabcYcaSiabdchaWnaaBaaameaacqWGJbWyaeqaaaWcbaWaaeWaceaacqWGobGtaiaawIcacaGLPaaacqGGSaalcqGHxiIkaaaaaOGaaCzcaiaaxMaadaqadiqaaiabikdaYiabigdaXaGaayjkaiaawMcaaaaa@5693@

**The monoisotopic mass as a function of the nominal mass **can be expressed by:

m(M)=λRC,pc(m),∗⋅m(N)(22)=mRC,pc(M),∗⋅m(N)mRC,pc(N),∗(23)=︸with Eq. 20 and 4mnone(M)⋅m(N)+∑n=0∞(1−pc)n⋅pc⋅(mRC(M)−mnone(M))⋅m¯(fc,n+n)mnone(N)+∑n=0∞(1−pc)n⋅pc⋅(mRC(N)−mnone(N))⋅m¯m(N)(fc,n+n)     (24)≈︸for m(N)≫m¯mnone(M)⋅m(N)mnone(N)+∑n=0∞(1−pc)n⋅pc⋅(mRC(M)−mnone(M))⋅m¯(fc,n+n)mnone(M)(25)
 MathType@MTEF@5@5@+=feaafiart1ev1aaatCvAUfKttLearuWrP9MDH5MBPbIqV92AaeXatLxBI9gBaebbnrfifHhDYfgasaacH8akY=wiFfYdH8Gipec8Eeeu0xXdbba9frFj0=OqFfea0dXdd9vqai=hGuQ8kuc9pgc9s8qqaq=dirpe0xb9q8qiLsFr0=vr0=vr0dc8meaabaqaciaacaGaaeqabaqabeGadaaakeGacaaWgaa8guaaaaqaeqaaaaaabaGaemyBa02aaWbaaSqabeaacqGGOaakcqWGnbqtcqGGPaqkaaaakeGabaqddiaaxMaacqGH9aqpaeGabaWNYJGaciab=T7aSnaaDaaaleaacqWGsbGudaWgaaadbaGaem4qameabeaaliabcYcaSiabdchaWnaaBaaameaacqWGJbWyaeqaaaWcbaGaeiikaGIaemyBa0MaeiykaKIaeiilaWIaey4fIOcaaOGaeyyXICTaemyBa02aaWbaaSqabeaacqGGOaakcqWGobGtcqGGPaqkaaaakeaadaqadiqaaiabikdaYiabikdaYaGaayjkaiaawMcaaaqaaaqaceaaLmGaaCzcaiabg2da9aqaamaalaaabaGaemyBa02aa0baaSqaaiabdkfasnaaBaaameaacqWGdbWqaeqaaSGaeiilaWIaemiCaa3aaSbaaWqaaiabdogaJbqabaaaleaacqGGOaakcqWGnbqtcqGGPaqkcqGGSaalcqGHxiIkaaGccqGHflY1cqWGTbqBdaahaaWcbeqaaiabcIcaOiabd6eaojabcMcaPaaaaOqaaiabd2gaTnaaDaaaleaacqWGsbGudaWgaaadbaGaem4qameabeaaliabcYcaSiabdchaWnaaBaaameaacqWGJbWyaeqaaaWcbaGaeiikaGIaemOta4KaeiykaKIaeiilaWIaey4fIOcaaaaaaOqaamaabmGabaGaeGOmaiJaeG4mamdacaGLOaGaayzkaaaabaaabaWaaGbaaeaacqGH9aqpaSqaaiabbEha3jabbMgaPjabbsha0jabbIgaOjabbccaGiabbweafjabbghaXjabb6caUiabbccaGiabbkdaYiabbcdaWiabbccaGiabbggaHjabb6gaUjabbsgaKjabbccaGiabbsda0aGccaGL44paaeaadaWcaaqaaiabd2gaTnaaDaaaleaacqWGUbGBcqWGVbWBcqWGUbGBcqWGLbqzaeaadaqadiqaaiabd2eanbGaayjkaiaawMcaaaaakiabgwSixlabd2gaTnaaCaaaleqabaWaaeWaceaacqWGobGtaiaawIcacaGLPaaaaaGccqGHRaWkdaaeWaqaamaabmGabaGaeGymaeJaeyOeI0IaemiCaa3aaSbaaSqaaiabdogaJbqabaaakiaawIcacaGLPaaadaahaaWcbeqaaiabd6gaUbaakiabgwSixlabdchaWnaaBaaaleaacqWGJbWyaeqaaOGaeyyXIC9aaeWaceaacqWGTbqBdaqhaaWcbaGaemOuai1aaSbaaWqaaiabdoeadbqabaaaleaadaqadiqaaiabd2eanbGaayjkaiaawMcaaaaakiabgkHiTiabd2gaTnaaDaaaleaacqWGUbGBcqWGVbWBcqWGUbGBcqWGLbqzaeaadaqadiqaaiabd2eanbGaayjkaiaawMcaaaaaaOGaayjkaiaawMcaaiabgwSixlqbd2gaTzaaraWaaeWaceaacqWGMbGzdaWgaaWcbaGaem4yamMaeiilaWIaemOBa4gabeaakiabgUcaRiabd6gaUbGaayjkaiaawMcaaaWcbaGaemOBa4Maeyypa0JaeGimaadabaGaeyOhIukaniabggHiLdaakeaacqWGTbqBdaqhaaWcbaGaemOBa4Maem4Ba8MaemOBa4MaemyzaugabaWaaeWaceaacqWGobGtaiaawIcacaGLPaaaaaGccqGHRaWkdaaeWaqaamaabmGabaGaeGymaeJaeyOeI0IaemiCaa3aaSbaaSqaaiabdogaJbqabaaakiaawIcacaGLPaaadaahaaWcbeqaaiabd6gaUbaakiabgwSixlabdchaWnaaBaaaleaacqWGJbWyaeqaaOGaeyyXIC9aaeWaceaacqWGTbqBdaqhaaWcbaGaemOuai1aaSbaaWqaaiabdoeadbqabaaaleaadaqadiqaaiabd6eaobGaayjkaiaawMcaaaaakiabgkHiTiabd2gaTnaaDaaaleaacqWGUbGBcqWGVbWBcqWGUbGBcqWGLbqzaeaadaqadiqaaiabd6eaobGaayjkaiaawMcaaaaaaOGaayjkaiaawMcaaiabgwSixpaalaaabaGafmyBa0MbaebaaeaacqWGTbqBdaahaaWcbeqaamaabmGabaGaemOta4eacaGLOaGaayzkaaaaaaaakmaabmGabaGaemOzay2aaSbaaSqaaiabdogaJjabcYcaSiabd6gaUbqabaGccqGHRaWkcqWGUbGBaiaawIcacaGLPaaaaSqaaiabd6gaUjabg2da9iabicdaWaqaaiabg6HiLcqdcqGHris5aaaakiaaxMaacaWLjaaabaWaaeWaceaacqaIYaGmcqaI0aanaiaawIcacaGLPaaaaeaaaeGabaq=aiaaxMaadaagaaqaaiabgIKi7cWcbaGaeeOzayMaee4Ba8MaeeOCaiNaeeiiaaIaemyBa02aaWbaaWqabeaadaqadiqaaiabd6eaobGaayjkaiaawMcaaaaaliablUMi=iqbd2gaTzaaraaakiaawIJ=aaqaamaalaaabaGaemyBa02aa0baaSqaaiabd6gaUjabd+gaVjabd6gaUjabdwgaLbqaaiabcIcaOiabd2eanjabcMcaPaaakiabgwSixlabd2gaTnaaCaaaleqabaGaeiikaGIaemOta4KaeiykaKcaaaGcbaGaemyBa02aa0baaSqaaiabd6gaUjabd+gaVjabd6gaUjabdwgaLbqaaiabcIcaOiabd6eaojabcMcaPaaaaaGccqGHRaWkdaWcaaqaamaaqadabaWaaeWaceaacqaIXaqmcqGHsislcqWGWbaCdaWgaaWcbaGaem4yamgabeaaaOGaayjkaiaawMcaamaaCaaaleqabaGaemOBa4gaaOGaeyyXICTaemiCaa3aaSbaaSqaaiabdogaJbqabaGccqGHflY1cqGGOaakcqWGTbqBdaqhaaWcbaGaemOuai1aaSbaaWqaaiabdoeadbqabaaaleaacqGGOaakcqWGnbqtcqGGPaqkaaGccqGHsislcqWGTbqBdaqhaaWcbaGaemOBa4Maem4Ba8MaemOBa4MaemyzaugabaGaeiikaGIaemyta0KaeiykaKcaaaqaaiabd6gaUjabg2da9iabicdaWaqaaiabg6HiLcqdcqGHris5aOGaeiykaKIaeyyXICTafmyBa0MbaebadaqadiqaaiabdAgaMnaaBaaaleaacqWGJbWycqGGSaalcqWGUbGBaeqaaOGaey4kaSIaemOBa4gacaGLOaGaayzkaaaabaGaemyBa02aa0baaSqaaiabd6gaUjabd+gaVjabd6gaUjabdwgaLbqaaiabcIcaOiabd2eanjabcMcaPaaaaaaakeaadaqadiqaaiabikdaYiabiwda1aGaayjkaiaawMcaaaaaaaa@8A55@

**Figure 3:**
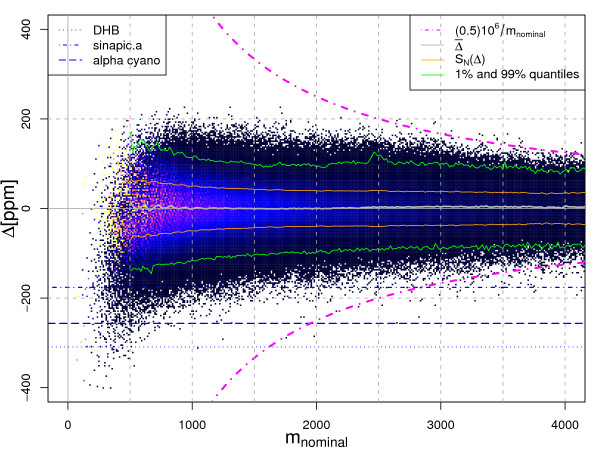
Deviation Δ^*ppm *^of peptide masses from mass cluster centres predicted using the Equation 24 in parts per million [ppm]. Gray line – moving average of Δ^*ppm*^. Orange lines – Standard deviation of Δ^*ppm*^, Green lines – 1% and 99% Quantile computed for mass windows having a size of 15*Da *and covering the mass range. Magenta dot dashed line – maximum possible deviation from cluster centre, which can be assigned to the true cluster centre using the Equation 30. Horizontal dotted blue line – distance of *DHB *(2,5-dihydroxybenzoic acid) matrix clusters from the peptide mass cluster centres; dashed line – distance of *alphacyano *(alpha-Cyano-4-hydroxycinnamic acid) clusters from the peptide mass cluster centres; distance of *sinapicacid *(3,5-Dimethoxy-4-hydroxycinnamic acid) clusters from peptide mass cluster centres.

This equation represents our final model of the peptide mass cluster centres. To illustrate the accuracy of the prediction we computed the residuals Δ between the monoisotopic masses of the *in silico *database digest and the cluster centres predicted by Equation 24. Figure 3 shows the relative residuals Δ^*ppm*^(*m*) = Δ(*m*)/*m*·10^6^, in parts per million. The grey line shows the moving average of the residuals Δ^*ppm*^(*m*) computed for a window of 15*Da*.

**Figure 4:**
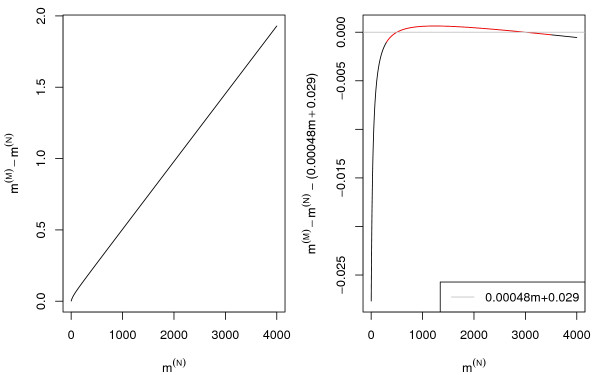
The monoisotopic mass as an function of the nominal mass. Left panel : *m*^(*M*) ^- *m*^(*N*) ^= (λRC,pc(m),∗
 MathType@MTEF@5@5@+=feaafiart1ev1aaatCvAUfKttLearuWrP9MDH5MBPbIqV92AaeXatLxBI9gBaebbnrfifHhDYfgasaacH8akY=wiFfYdH8Gipec8Eeeu0xXdbba9frFj0=OqFfea0dXdd9vqai=hGuQ8kuc9pgc9s8qqaq=dirpe0xb9q8qiLsFr0=vr0=vr0dc8meaabaqaciaacaGaaeqabaqabeGadaaakeaaiiGacqWF7oaBdaqhaaWcbaGaemOuai1aaSbaaWqaaiabdoeadbqabaWccqGGSaalcqWGWbaCdaWgaaadbaGaem4yamgabeaaaSqaaiabcIcaOiabd2gaTjabcMcaPiabcYcaSiabgEHiQaaaaaa@39BC@ - 1)·*m*^(*N*) ^Right panel : Difference between (λRC,pc(m),∗
 MathType@MTEF@5@5@+=feaafiart1ev1aaatCvAUfKttLearuWrP9MDH5MBPbIqV92AaeXatLxBI9gBaebbnrfifHhDYfgasaacH8akY=wiFfYdH8Gipec8Eeeu0xXdbba9frFj0=OqFfea0dXdd9vqai=hGuQ8kuc9pgc9s8qqaq=dirpe0xb9q8qiLsFr0=vr0=vr0dc8meaabaqaciaacaGaaeqabaqabeGadaaakeaaiiGacqWF7oaBdaqhaaWcbaGaemOuai1aaSbaaWqaaiabdoeadbqabaWccqGGSaalcqWGWbaCdaWgaaadbaGaem4yamgabeaaaSqaaiabcIcaOiabd2gaTjabcMcaPiabcYcaSiabgEHiQaaaaaa@39BC@ - 1) *m*^(*N*) ^and 0.00048 *m*^(*N*) ^+ 0.029.

Figure 4, panel A, shows the difference between nominal and monoisotopic mass (*m*^(*M*) ^- *m*^(*N*)^) where *m*^(*M*) ^was predicted using the model of Equation 24. We observed that *m*^(*M*) ^- *m*^(*N*) ^∝ *m*^(*N*) ^is approximately a straight line for the mass range greater than 500*Da*. By using the predicted monoisotopic mass *m*^(*M*) ^at *m*^(*N*) ^= 500 and at *m*^(*N*) ^= 3000 we determined the slope:

c1=3000⋅λRC,pc(3000),∗−500⋅λRC,pc(500),∗3000−500=1.000482,     (26)
 MathType@MTEF@5@5@+=feaafiart1ev1aaatCvAUfKttLearuWrP9MDH5MBPbIqV92AaeXatLxBI9gBaebbnrfifHhDYfgasaacH8akY=wiFfYdH8Gipec8Eeeu0xXdbba9frFj0=OqFfea0dXdd9vqai=hGuQ8kuc9pgc9s8qqaq=dirpe0xb9q8qiLsFr0=vr0=vr0dc8meaabaqaciaacaGaaeqabaqabeGadaaakeaacqWGJbWydaWgaaWcbaGaeGymaedabeaakiabg2da9maalaaabaGaeG4mamJaeGimaaJaeGimaaJaeGimaaJaeyyXICncciGae83UdW2aa0baaSqaaiabdkfasnaaBaaameaacqWGdbWqaeqaaSGaeiilaWIaemiCaa3aaSbaaWqaaiabdogaJbqabaaaleaadaqadiqaaiabiodaZiabicdaWiabicdaWiabicdaWaGaayjkaiaawMcaaiabcYcaSiabgEHiQaaakiabgkHiTiabiwda1iabicdaWiabicdaWiabgwSixlab=T7aSnaaDaaaleaacqWGsbGudaWgaaadbaGaem4qameabeaaliabcYcaSiabdchaWnaaBaaameaacqWGJbWyaeqaaaWcbaWaaeWaceaacqaI1aqncqaIWaamcqaIWaamaiaawIcacaGLPaaacqGGSaalcqGHxiIkaaaakeaacqaIZaWmcqaIWaamcqaIWaamcqaIWaamcqGHsislcqaI1aqncqaIWaamcqaIWaamaaGaeyypa0JaeGymaeJaeiOla4IaeGimaaJaeGimaaJaeGimaaJaeGinaqJaeGioaGJaeGOmaiJaeiilaWIaaCzcaiaaxMaadaqadiqaaiabikdaYiabiAda2aGaayjkaiaawMcaaaaa@6F94@

and intercept coefficient

c0=500⋅(λRC,pc(500),∗−1)−c1⋅500=0.029.     (27)
 MathType@MTEF@5@5@+=feaafiart1ev1aaatCvAUfKttLearuWrP9MDH5MBPbIqV92AaeXatLxBI9gBaebbnrfifHhDYfgasaacH8akY=wiFfYdH8Gipec8Eeeu0xXdbba9frFj0=OqFfea0dXdd9vqai=hGuQ8kuc9pgc9s8qqaq=dirpe0xb9q8qiLsFr0=vr0=vr0dc8meaabaqaciaacaGaaeqabaqabeGadaaakeaacqWGJbWydaWgaaWcbaGaeGimaadabeaakiabg2da9iabiwda1iabicdaWiabicdaWiabgwSixpaabmGabaacciGae83UdW2aa0baaSqaaiabdkfasnaaBaaameaacqWGdbWqaeqaaSGaeiilaWIaemiCaa3aaSbaaWqaaiabdogaJbqabaaaleaadaqadiqaaiabiwda1iabicdaWiabicdaWaGaayjkaiaawMcaaiabcYcaSiabgEHiQaaakiabgkHiTiabigdaXaGaayjkaiaawMcaaiabgkHiTiabdogaJnaaBaaaleaacqaIXaqmaeqaaOGaeyyXICTaeGynauJaeGimaaJaeGimaaJaeyypa0JaeGimaaJaeiOla4IaeGimaaJaeGOmaiJaeGyoaKJaeiOla4IaaCzcaiaaxMaadaqadiqaaiabikdaYiabiEda3aGaayjkaiaawMcaaaaa@5AE8@

These coefficients are in good agreement with the slope and intercept determined by linear regression for the *in silico *sequence database digest (Figure 1).

Furthermore, we observed that the intercept *c*_0 _will be positive if mRC
 MathType@MTEF@5@5@+=feaafiart1ev1aaatCvAUfKttLearuWrP9MDH5MBPbIqV92AaeXatLxBI9gBaebbnrfifHhDYfgasaacH8akY=wiFfYdH8Gipec8Eeeu0xXdbba9frFj0=OqFfea0dXdd9vqai=hGuQ8kuc9pgc9s8qqaq=dirpe0xb9q8qiLsFr0=vr0=vr0dc8meaabaqaciaacaGaaeqabaqabeGadaaakeaacqWGTbqBdaWgaaWcbaGaemOuai1aaSbaaWqaaiabdoeadbqabaaaleqaaaaa@30AF@ > *m*_*none*_, zero or negative otherwise. The slope *c*_1 _equals *λ*_*none *_= mnone(M)mnone(N)
 MathType@MTEF@5@5@+=feaafiart1ev1aaatCvAUfKttLearuWrP9MDH5MBPbIqV92AaeXatLxBI9gBaebbnrfifHhDYfgasaacH8akY=wiFfYdH8Gipec8Eeeu0xXdbba9frFj0=OqFfea0dXdd9vqai=hGuQ8kuc9pgc9s8qqaq=dirpe0xb9q8qiLsFr0=vr0=vr0dc8meaabaqaciaacaGaaeqabaqabeGadaaakeaadaWcaaqaaiabd2gaTnaaDaaaleaacqWGUbGBcqWGVbWBcqWGUbGBcqWGLbqzaeaadaqadiqaaiabd2eanbGaayjkaiaawMcaaaaaaOqaaiabd2gaTnaaDaaaleaacqWGUbGBcqWGVbWBcqWGUbGBcqWGLbqzaeaadaqadiqaaiabd6eaobGaayjkaiaawMcaaaaaaaaaaa@404C@, for large *m*^(*N*)^, because the frequency of the cleavage site residues *R*_*C *_decreases with increasing peptide length:

lim⁡|Pep|→∞fm,n∝lim⁡mpep→∞(n+1)m¯m(N)=0.
 MathType@MTEF@5@5@+=feaafiart1ev1aaatCvAUfKttLearuWrP9MDH5MBPbIqV92AaeXatLxBI9gBaebbnrfifHhDYfgasaacH8akY=wiFfYdH8Gipec8Eeeu0xXdbba9frFj0=OqFfea0dXdd9vqai=hGuQ8kuc9pgc9s8qqaq=dirpe0xb9q8qiLsFr0=vr0=vr0dc8meaabaqaciaacaGaaeqabaqabeGadaaakeaadaWfqaqaaiGbcYgaSjabcMgaPjabc2gaTbWcbaGaeiiFaWNaemiuaaLaemyzauMaemiCaaNaeiiFaWNaeyOKH4QaeyOhIukabeaakiabdAgaMnaaBaaaleaacqWGTbqBcqGGSaalcqWGUbGBaeqaaOGaeyyhIu7aaCbeaeaacyGGSbaBcqGGPbqAcqGGTbqBaSqaaiabd2gaTnaaBaaameaacqWGWbaCcqWGLbqzcqWGWbaCaeqaaSGaeyOKH4QaeyOhIukabeaakmaalaaabaWaaeWaceaacqWGUbGBcqGHRaWkcqaIXaqmaiaawIcacaGLPaaacuWGTbqBgaqeaaqaaiabd2gaTnaaCaaaleqabaWaaeWaceaacqWGobGtaiaawIcacaGLPaaaaaaaaOGaeyypa0JaeGimaaJaeiOla4caaa@5CF1@

Figure 4, panel B, displays the difference between the line (*c*_1 _+ 1)·*m*^(*M*) ^+ *c*_0 _and the prediction made using Equation 3. For the mass range *m *∈ (500, 4000) where peptide masses for peptide mass fingerprinting are acquired this difference is minimal.

The coefficients *c*_0 _and *c*_1 _do not depend on the mass of the peptides. Due to this feature, we are going to use the affine model *c*_1_*m*^(*N*) ^+ *c*_0 _to predict the peptide mass cluster centres in the applications discussed later. This simplified model is also in agreement with the affine model (Equation 3), which has been fitted by linear regression to the *in silico *database digest in order to explain the dependency of the peptide mass cluster centres on the nominal mass.

### Error of the model

**Figure 5:**
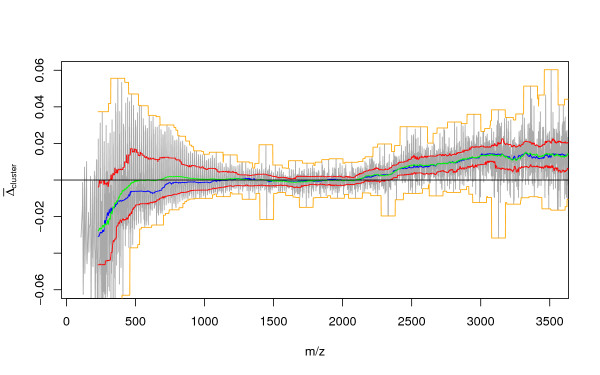
Difference between cluster centre computed for the *in silico *database digest and the cluster centre location predicted by the model (Equation 24). Orange lines – minimum and maximum, red lines – first and third quartile, green – mean, blue – median of the differences computed for a moving window of 100*Da*.

Combinatorial restrictions may cause significant differences between the linear prediction of the model (Equation 24) introduced and the actual location of the cluster centre. To asses this error we first computed the location of the cluster centres (average of all monoisotopic masses in cluster) of the *in silico *database digest, and afterwards determined the difference to the cluster centre location predicted by model of Equation 24. This difference Δ¯
 MathType@MTEF@5@5@+=feaafiart1ev1aaatCvAUfKttLearuWrP9MDH5MBPbIqV92AaeXatLxBI9gBaebbnrfifHhDYfgasaacH8akY=wiFfYdH8Gipec8Eeeu0xXdbba9frFj0=OqFfea0dXdd9vqai=hGuQ8kuc9pgc9s8qqaq=dirpe0xb9q8qiLsFr0=vr0=vr0dc8meaabaqaciaacaGaaeqabaqabeGadaaakeaacuqHuoargaqeaaaa@2E2A@(*cluster*) is shown in Figure 5.

For a moving window of 100*Da *we computed the maximum and minimum (orange), third and first quartile (red), median (blue) and mean(gree) of Δ¯
 MathType@MTEF@5@5@+=feaafiart1ev1aaatCvAUfKttLearuWrP9MDH5MBPbIqV92AaeXatLxBI9gBaebbnrfifHhDYfgasaacH8akY=wiFfYdH8Gipec8Eeeu0xXdbba9frFj0=OqFfea0dXdd9vqai=hGuQ8kuc9pgc9s8qqaq=dirpe0xb9q8qiLsFr0=vr0=vr0dc8meaabaqaciaacaGaaeqabaqabeGadaaakeaacuqHuoargaqeaaaa@2E2A@(*cluster*). The combinatorial restriction decreases with increasing mass and for peptide masses greater than 1000*Da *it is negligible. However, Δ¯
 MathType@MTEF@5@5@+=feaafiart1ev1aaatCvAUfKttLearuWrP9MDH5MBPbIqV92AaeXatLxBI9gBaebbnrfifHhDYfgasaacH8akY=wiFfYdH8Gipec8Eeeu0xXdbba9frFj0=OqFfea0dXdd9vqai=hGuQ8kuc9pgc9s8qqaq=dirpe0xb9q8qiLsFr0=vr0=vr0dc8meaabaqaciaacaGaaeqabaqabeGadaaakeaacuqHuoargaqeaaaa@2E2A@(*cluster*) increases again for masses greater than 2500*Da *because peptide masses may deviate more strongly from the cluster centres and furthermore much fewer long peptides are generated.

### The type of distribution around the cluster centres

In order to remove non-peptide peaks prior to database search, filtering thresholds have to be chosen. In Figure 3 the orange line visualises the standard deviation while the green lines show the 1% and 99% quantiles of Δ^*ppm*^(*m*) = Δ(*m*)/*m*·10^6 ^computed for a mass window of 15*Da*. In addition the dotted, dashed, and dot dashed line show the deviation Δ^*ppm*^(*m*), at which clusters of mass spectrometric matrices are expected.

**Figure 6:**
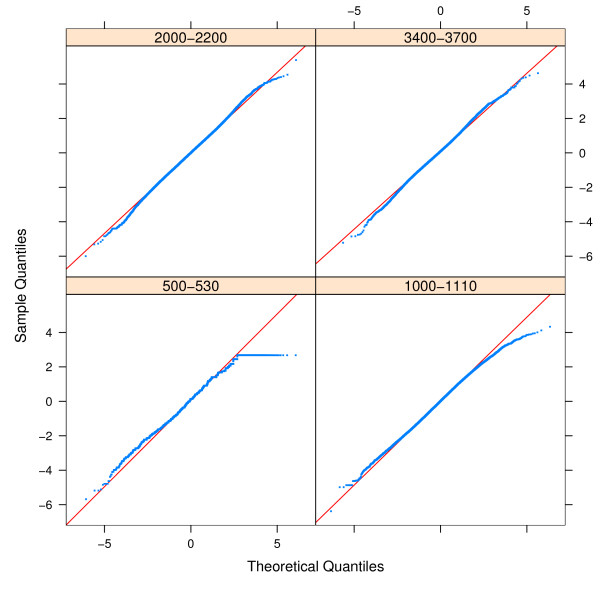
qqplot – of Δ^*ppm *^= *m*_*m *_- *c*_1_·*m*_*N *_- *c*_0 _versus the t-distribution with 19 degrees of freedom for four mass ranges *m *∈ (500 – 530), *m *∈ (1000 – 1110), *m *∈ (2000 – 2200)and *m *∈ (3400 – 3700).

The standard deviation of Δ^*ppm*^(*m*) is symmetric and does not change for *m *> 1500. We were interested to determine the distribution of Δ^*ppm *^around the peptide mass cluster centres. To determine the type of distribution we use qqplots [28] shown in Figure 6. We compared the distribution of the residues Δ^*ppm*^(*m*), observed for four different mass windows (*m *∈ (500 – 530), *m *∈ (1000 – 1110), *m *∈ (2000 – 2200) and *m *∈ (3400 – 3700)) with the normal distribution and t-distributions with various degrees of freedom. The t-distribution with degrees of freedom *μ *∈ (15, 25) is a good approximation of the empirical distribution of Δ^*ppm *^for masses > 2000,.

### Sensitivity analysis

The input parameters to the model of the peptide mass cluster centres included:

• *f*_*i *_– frequencies of the amino acids.

• cleavage specificity of the protease *R*_*C*_

• |*P*| – Protein length

• *p*_*c *_– cleavage probability

To examine how the output of the model is influenced by these factors we varied the protein length |*P*| in steps of 100 from 300 to 800 amino acids per protein. We determined the amino acid frequencies *f*_*i *_for 9 sequence databases (cf. Methods) and used them as inputs to the model. Furthermore, six cleavage specificities (shown in Table 3) were examined and the cleavage probability *p*_*c *_was changed from 0.4 to 1 in increments of 0.2.

**Table 3:** Cleavage sites of proteolytic enzymes [36]

	Enzyme	*R*_*C*_
1	Trypsin/P	K,R/P
2	Arg.C	R/P
3	CNBR + Trypsin	F, Y, M
4	Lys-C	K/P
5	PepsinA	F, L
6	CNBr	M

**Figure 7:**
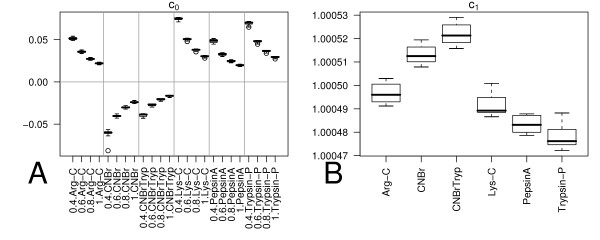
Panel A – Box plots of the intercept coefficient *c*_0 _(Equation 27) itemised according the cleavage specificity and cleavage probability. Panel B – Box plots of the slope coefficient *c*_1 _(Equation 26) itemised according the cleavage specificity.

The box-plots, of Figure 7, Panel A demonstrate that the values of the intercept coefficient *c*_0 _(Equation 27) mainly depend on the cleavage probability *p*_*c *_and on the cleavage specificity of the proteolytic enzyme. The relatively small height of the boxes indicates that the differences in amino acid frequencies *f*_*i *_for the databases examined, and the average protein length |*P*| have a negligible effect on the intercept coefficient. The slope coefficient *c*_1 _(see Equation 26) depends only on the cleavage site specificities of the proteolytic enzyme and the amino acid frequencies *f*. The box-plots 7 Panel B show that the model output is highly sensitive to the cleavage specificity of the proteolytic enzyme.

### A measure of distance to cluster centres

Given an experimentally determined *m*_*M *_we were interested to estimate the deviation Δ from the closest predicted cluster centre. The model of the monoisotopic mass is:

*c*_0 _+ *c*_1_·*m*_*N *_+ Δ = *m*_*M*_,     (28)

where *c*_0_, *c*_1 _can be obtained using the Equations 27 and 26, *m*_*N *_is the nominal mass (an integer).

Therefore, for a given *m*_*M*_, *c*_0 _and *c*_1 _we can determine the deviation Δ from the closest cluster centre of *smaller *mass by using the modulo operator as suggested by Wool and Smilansky [10]:

(*m*_*M *_- *c*_0_)(mod*c*_1_) = (*c*_1_·*m *+ Δ)(mod*c*_1_) = Δ.     (29)

However, in order to determine the distance to the closest cluster centre we considered two cases:

Δλ(mi,0)={(mi−c0)(mod⁡c1)if(mi−c0)(mod⁡λnone)<0.5−1+(mi−c0)(mod⁡c1)otherwise.     (30)
 MathType@MTEF@5@5@+=feaafiart1ev1aaatCvAUfKttLearuWrP9MDH5MBPbIqV92AaeXatLxBI9gBaebbnrfifHhDYfgasaacH8akY=wiFfYdH8Gipec8Eeeu0xXdbba9frFj0=OqFfea0dXdd9vqai=hGuQ8kuc9pgc9s8qqaq=dirpe0xb9q8qiLsFr0=vr0=vr0dc8meaabaqaciaacaGaaeqabaqabeGadaaakeaacqGHuoardaWgaaWcbaacciGae83UdWgabeaakmaabmGabaGaemyBa02aaSbaaSqaaiabdMgaPbqabaGccqGGSaalcqaIWaamaiaawIcacaGLPaaacqGH9aqpdaGabeqaauaabaqacmaaaeaadaqadiqaaiabd2gaTnaaBaaaleaacqWGPbqAaeqaaOGaeyOeI0Iaem4yam2aaSbaaSqaaiabicdaWaqabaaakiaawIcacaGLPaaadaqadiqaaiGbc2gaTjabc+gaVjabcsgaKjabdogaJnaaBaaaleaacqaIXaqmaeqaaaGccaGLOaGaayzkaaaabaGaeeyAaKMaeeOzaygabaWaaeWaceaacqWGTbqBdaWgaaWcbaGaemyAaKgabeaakiabgkHiTiabdogaJnaaBaaaleaacqaIWaamaeqaaaGccaGLOaGaayzkaaWaaeWaceaacyGGTbqBcqGGVbWBcqGGKbazcqWF7oaBdaWgaaWcbaGaemOBa4Maem4Ba8MaemOBa4MaemyzaugabeaaaOGaayjkaiaawMcaaiabgYda8iabicdaWiabc6caUiabiwda1aqaaiabgkHiTiabigdaXiabgUcaRmaabmGabaGaemyBa02aaSbaaSqaaiabdMgaPbqabaGccqGHsislcqWGJbWydaWgaaWcbaGaeGimaadabeaaaOGaayjkaiaawMcaamaabmGabaGagiyBa0Maei4Ba8MaeiizaqMaem4yam2aaSbaaSqaaiabigdaXaqabaaakiaawIcacaGLPaaaaeaacqqGVbWBcqqG0baDcqqGObaAcqqGLbqzcqqGYbGCcqqG3bWDcqqGPbqAcqqGZbWCcqqGLbqzcqGGUaGlaeaaaaGaaCzcaiaaxMaadaqadiqaaiabiodaZiabicdaWaGaayjkaiaawMcaaaGaay5Eaaaaaa@88A4@

The units of Δ_*λ*_(*m*_*i*_, 0) are in [*m*/*z*]. The magenta dot dashed curves in Figure 3 indicate the maximum detectable distance from cluster centres in ppm (±0.5*Da*/*m*·10^6^[*ppm*]). Deviations from the cluster centres outside the range enclosed by these two curves are assigned to the wrong cluster. In case of theoretical peptide masses and experimental masses calibrated to high precision, such distances are observed only for masses greater than 2500*Da*. Fortunately, the majority of tryptic peptide masses detected in a mass spectrometric peptide fingerprint experiment are below this mass.

### Applications

#### Linear regression on peptide mass rule LR/PR

The limitations of calibration methods based on the property of peptide mass clustering are a mass accuracy of only 0.2*Da*, its sensitivity to non-peptide peaks in the spectra, and that it completely fails if the number of peptide peaks in the peak list is small [10,14,19]. Hence, in practice, the method is used to confirm the results of internal calibration only [14,29]. However, the advantage of the calibration methods based on the property of peptide mass clustering, over other calibration methods [12], is that no internal or external calibrants are required in order to calibrate the peptide mass lists.

We propose here a novel method for the calibration of PMF data, based on robust linear regression and the distance measure introduced in the Equation 30. To determine the slope of the mass measurement error we computed the deviation from the peptide mass rule for every pair of peak masses (*m*_*i*_, *m*_*j*_) within a peak-list, employing the following equation:

Δλ(mi,mj)={|mi−mj|(mod⁡λnone)if|mi−mj|(mod⁡λnone)<0.5−1+(|mi−mj|(mod⁡λnone))otherwise.     (31)
 MathType@MTEF@5@5@+=feaafiart1ev1aaatCvAUfKttLearuWrP9MDH5MBPbIqV92AaeXatLxBI9gBaebbnrfifHhDYfgasaacH8akY=wiFfYdH8Gipec8Eeeu0xXdbba9frFj0=OqFfea0dXdd9vqai=hGuQ8kuc9pgc9s8qqaq=dirpe0xb9q8qiLsFr0=vr0=vr0dc8meaabaqaciaacaGaaeqabaqabeGadaaakeaacqGHuoardaWgaaWcbaacciGae83UdWgabeaakmaabmGabaGaemyBa02aaSbaaSqaaiabdMgaPbqabaGccqGGSaalcqWGTbqBdaWgaaWcbaGaemOAaOgabeaaaOGaayjkaiaawMcaaiabg2da9maaceqabaqbaeaabiWaaaqaaiabcYha8jabd2gaTnaaBaaaleaacqWGPbqAaeqaaOGaeyOeI0IaemyBa02aaSbaaSqaaiabdQgaQbqabaGccqGG8baFdaqadiqaaiGbc2gaTjabc+gaVjabcsgaKjab=T7aSnaaBaaaleaacqWGUbGBcqWGVbWBcqWGUbGBcqWGLbqzaeqaaaGccaGLOaGaayzkaaaabaGaeeyAaKMaeeOzaygabaGaeiiFaWNaemyBa02aaSbaaSqaaiabdMgaPbqabaGccqGHsislcqWGTbqBdaWgaaWcbaGaemOAaOgabeaakiabcYha8naabmGabaGagiyBa0Maei4Ba8MaeiizaqMae83UdW2aaSbaaSqaaiabd6gaUjabd+gaVjabd6gaUjabdwgaLbqabaaakiaawIcacaGLPaaacqGH8aapcqaIWaamcqGGUaGlcqaI1aqnaeaacqGHsislcqaIXaqmcqGHRaWkdaqadiqaaiabcYha8jabd2gaTnaaBaaaleaacqWGPbqAaeqaaOGaeyOeI0IaemyBa02aaSbaaSqaaiabdQgaQbqabaGccqGG8baFdaqadiqaaiGbc2gaTjabc+gaVjabcsgaKjab=T7aSnaaBaaaleaacqWGUbGBcqWGVbWBcqWGUbGBcqWGLbqzaeqaaaGccaGLOaGaayzkaaaacaGLOaGaayzkaaaabaGaee4Ba8MaeeiDaqNaeeiAaGMaeeyzauMaeeOCaiNaee4DaCNaeeyAaKMaee4CamNaeeyzauMaeeOla4cabaaaaiaaxMaacaWLjaWaaeWaceaacqaIZaWmcqaIXaqmaiaawIcacaGLPaaaaiaawUhaaaaa@9C08@

**Figure 8:**
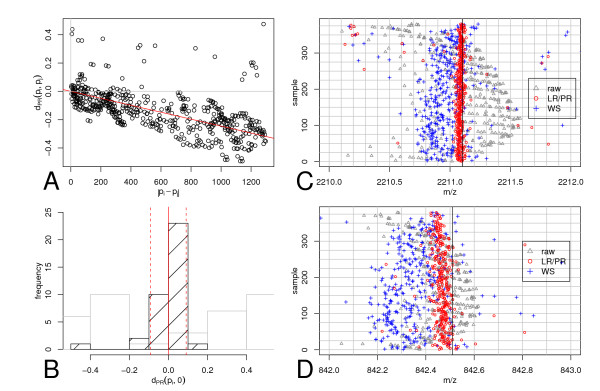
Principle and results of linear regression on peptide rule *LR/PR *calibration. Panel A: Scatter-plot of Δ_*PR *_(*m*_*i*_, *m*_*j*_) (Equation 31) in dependence of Δ_*d *_= |*m*_*i *_- *m*_*j*_|. The slope, obtained by robust regression, is shown by the red line. Panel B: Histogram (black with diagonals) of *d*_*PR*_(*m*_*i*_, 0). The continuous vertical red line denotes the average (d¯
 MathType@MTEF@5@5@+=feaafiart1ev1aaatCvAUfKttLearuWrP9MDH5MBPbIqV92AaeXatLxBI9gBaebbnrfifHhDYfgasaacH8akY=wiFfYdH8Gipec8Eeeu0xXdbba9frFj0=OqFfea0dXdd9vqai=hGuQ8kuc9pgc9s8qqaq=dirpe0xb9q8qiLsFr0=vr0=vr0dc8meaabaqaciaacaGaaeqabaqabeGadaaakeaacuWGKbazgaqeaaaa@2E15@_*PR*_(*m*_*i*_, 0)) and the dotted vertical lines denote (d¯
 MathType@MTEF@5@5@+=feaafiart1ev1aaatCvAUfKttLearuWrP9MDH5MBPbIqV92AaeXatLxBI9gBaebbnrfifHhDYfgasaacH8akY=wiFfYdH8Gipec8Eeeu0xXdbba9frFj0=OqFfea0dXdd9vqai=hGuQ8kuc9pgc9s8qqaq=dirpe0xb9q8qiLsFr0=vr0=vr0dc8meaabaqaciaacaGaaeqabaqabeGadaaakeaacuWGKbazgaqeaaaa@2E15@_*PR*_(*m*_*i*_, 0) ± *S*_*N*_. The histogram in gray is showing the distribution of (*d*_*PR*_(*m*_*i*_, 0) previous to removing the slope error (see text). Panel C & D: Strip-charts of the data-set for a mass range of 2210 – 2212*Da *and 842 – 843*Da*, including the tryptic autolysis peaks 842.508*Da *and 2211.100*Da*. Gray triangles – raw data; blue "+" – Wool Smilansky algorithm (cf. Appendix); red "o" – LR/RP algorithm for tryptic peaks .

Figure 8 left top panel shows the distance Δ_*λ*_(*m*_*i*_, *m*_*j*_) (Equation 31) as a function of Δ_*d *_= |*m*_*i *_- *m*_*j*_|, computed for all pairs (*m*_*i*_, *m*_*j*_) ∈ peak-list, which adhere to the additional constraint that Δ_*d *_= |*m*_*i *_- *m*_*j*_| <*m*_*max*_. This constraint is necessary because the measure Δ_*λ *_is only able to assign deviation smaller than 0.5*Da *to the correct cluster centre. For large values of Δ_*d*_, Δ_*λ *_increases, if *c*_1 _≠ 0 and assignments to wrong clusters may occur. If a systematic dependence of Δ_*λ *_on Δ_*d *_is observed it indicates a mass measurement error. We determined the slope c^
 MathType@MTEF@5@5@+=feaafiart1ev1aaatCvAUfKttLearuWrP9MDH5MBPbIqV92AaeXatLxBI9gBaebbnrfifHhDYfgasaacH8akY=wiFfYdH8Gipec8Eeeu0xXdbba9frFj0=OqFfea0dXdd9vqai=hGuQ8kuc9pgc9s8qqaq=dirpe0xb9q8qiLsFr0=vr0=vr0dc8meaabaqaciaacaGaaeqabaqabeGadaaakeaacuWGJbWygaqcaaaa@2E0B@_1 _using robust linear regression [30] with the intercept fixed at 0. To correct the peak-list masses we applied

*m*_*corrected *_= *m*_*experimental*_·(1 - c^
 MathType@MTEF@5@5@+=feaafiart1ev1aaatCvAUfKttLearuWrP9MDH5MBPbIqV92AaeXatLxBI9gBaebbnrfifHhDYfgasaacH8akY=wiFfYdH8Gipec8Eeeu0xXdbba9frFj0=OqFfea0dXdd9vqai=hGuQ8kuc9pgc9s8qqaq=dirpe0xb9q8qiLsFr0=vr0=vr0dc8meaabaqaciaacaGaaeqabaqabeGadaaakeaacuWGJbWygaqcaaaa@2E0B@_1_)

To determine the intercept coefficient of the mass measurement error we subsequently computed Δ_*λ*_(*m*_*corrected*_, 0) (using Equation 30), for all peak-list masses. Figure 8, Panel B shows the distribution of Δ_*λ*_(*m*_*i*_, 0) before correcting for the slope error (gray histogram) and afterwards (black histogram). The red vertical line indicates the mean Δ¯
 MathType@MTEF@5@5@+=feaafiart1ev1aaatCvAUfKttLearuWrP9MDH5MBPbIqV92AaeXatLxBI9gBaebbnrfifHhDYfgasaacH8akY=wiFfYdH8Gipec8Eeeu0xXdbba9frFj0=OqFfea0dXdd9vqai=hGuQ8kuc9pgc9s8qqaq=dirpe0xb9q8qiLsFr0=vr0=vr0dc8meaabaqaciaacaGaaeqabaqabeGadaaakeaacuqHuoargaqeaaaa@2E2A@_*λ*_(*m*_*i*_, 0), computed for the corrected data, which we used to approximate the intercept c^
 MathType@MTEF@5@5@+=feaafiart1ev1aaatCvAUfKttLearuWrP9MDH5MBPbIqV92AaeXatLxBI9gBaebbnrfifHhDYfgasaacH8akY=wiFfYdH8Gipec8Eeeu0xXdbba9frFj0=OqFfea0dXdd9vqai=hGuQ8kuc9pgc9s8qqaq=dirpe0xb9q8qiLsFr0=vr0=vr0dc8meaabaqaciaacaGaaeqabaqabeGadaaakeaacuWGJbWygaqcaaaa@2E0B@_0 _of the mass measurement error.

The strip charts (Figure 8, Panel C and D) visualises the experimental masses of two trypsin peptides 842.508*Da *and 2211.100*Da *observed in most of the samples of the dataset with 380 peak-lists. The result of LR/PR calibration (red circles) is compared with raw masses (gray triangles) and the output of the Wool and Smilansky calibration method (blue crosses). The LR/PR-method is able to calibrate mass spectrometric peak-lists to an accuracy of 0.1*Da*. This measurement accuracy surpasses the other published calibration methods [10,19] at least two-fold.

#### Filtering of non-peptide peaks using the peptide mass rule

**Figure 9:**
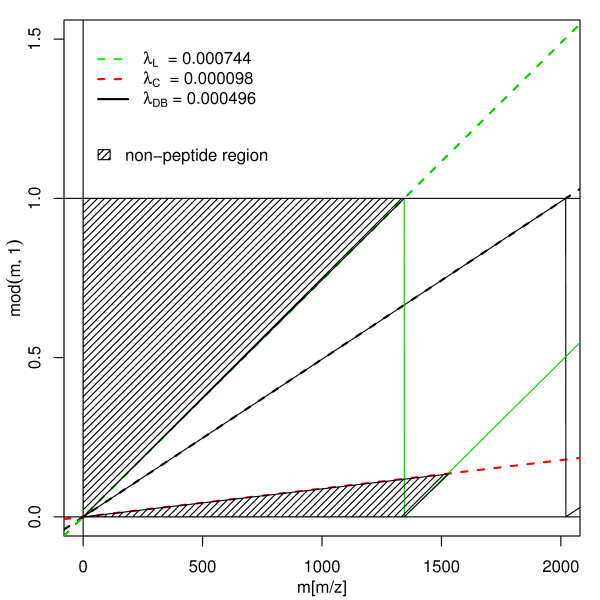
Schema of non-peptide mass filtering. Abscissae – peptide mass, ordinate – *m *mod 1, dashed region – non-peptide masses. Green line – decimal part of poly-(L(lys), I(ile)) peptide masses as a function of their mass. Red line – decimal part of poly-(C(cys)) peptide masses as function of their mass. Black line – Predicted cluster centres using the Equation 2.

Non-peptide peaks can be recognised according to their deviation from the cluster centres. The amino acids that have the most extreme *λ *values are I, L and K (because of their large fraction of Hydrogen H (1.007825) atoms) and C (Cysteine – because of the heavy sulfur atom S (31.97207)). If we plot the position after the decimal point given by *n*·(*λ*_*i *_- l)(modl) with *n *∈ ℕ, for *i *= *L *and *i *= *C*, and connect the points for readability purposes by a line (the red and green lines in Figure 9 respectively), we obtain the range enclosing any possible decimal point a theoretical peptide mass can have. If a mass with a decimal point lying in the dashed region is detected it can not be a peptide peak. For peptide peaks, the following inequalities hold:

-413[*ppm*] = (*λ*_*C *_- *λ*_*DB*_)·10^6 ^< Δ_*Δ*_(*m*, 0)·10^6^/*m *= Δλppm
 MathType@MTEF@5@5@+=feaafiart1ev1aaatCvAUfKttLearuWrP9MDH5MBPbIqV92AaeXatLxBI9gBaebbnrfifHhDYfgasaacH8akY=wiFfYdH8Gipec8Eeeu0xXdbba9frFj0=OqFfea0dXdd9vqai=hGuQ8kuc9pgc9s8qqaq=dirpe0xb9q8qiLsFr0=vr0=vr0dc8meaabaqaciaacaGaaeqabaqabeGadaaakeaacqGHuoardaqhaaWcbaacciGae83UdWgabaGaemiCaaNaemiCaaNaemyBa0gaaaaa@3430@ (*m*, 0) < (*λ*_*L *_- *λ*_*DB*_) = 241[*ppm*],     (32)

where *λ*_*DB *_= 1.000511 (Equation 2). We used the relative deviation of Δ^*ppm *^from the cluster centre in parts per million instead of using absolute values.

Figure 3 shows that only very short peptides approach the lower bound of -413*ppm*. This is due to the low frequency of Cysteine (C). The high frequencies of *K*, *L*, *I *(whose *λ *≈ 1.00074) mean that the theoretical upper bound of 241*ppm *can indeed be reached by some peptides with a mass of ≈ l000*Da*. Peptides of higher mass never approach the upper and lower theoretical bound due to the rapidly decreasing probability to consist of *K*, *L *or *I*, or of *C *only. The lines for the standard deviation of *S*_*N *_(orange lines) and of the 1% and 99% quantile (green lines) in Figure 3 indicate that it is an exceedingly rare event to encounter a peptide mass for which Δλppm
 MathType@MTEF@5@5@+=feaafiart1ev1aaatCvAUfKttLearuWrP9MDH5MBPbIqV92AaeXatLxBI9gBaebbnrfifHhDYfgasaacH8akY=wiFfYdH8Gipec8Eeeu0xXdbba9frFj0=OqFfea0dXdd9vqai=hGuQ8kuc9pgc9s8qqaq=dirpe0xb9q8qiLsFr0=vr0=vr0dc8meaabaqaciaacaGaaeqabaqabeGadaaakeaacqGHuoardaqhaaWcbaacciGae83UdWgabaGaemiCaaNaemiCaaNaemyBa0gaaaaa@3430@(*m*, 0) will deviate more than 200*ppm *from the peptide cluster centre predicted by our model. Therefore, we use 200*ppm *as a filtering threshold. An essential requirement, to apply this filtering method successfully is that peak-list must be calibrated to high precision [12].

**Figure 10:**
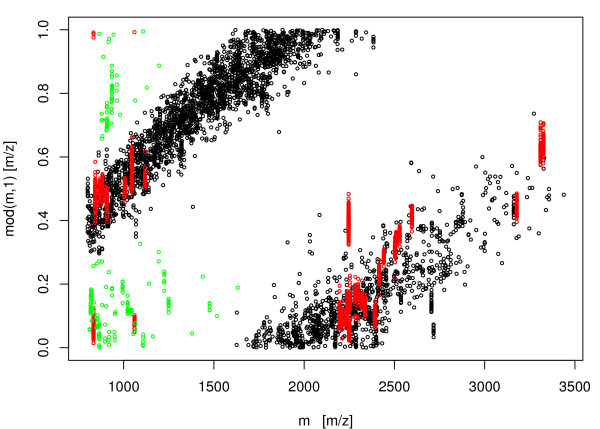
Scatter plot : abscissae – peptide mass *m*_*i*_, ordinate – *m*_*i*_*modλ *with *λ *= 1.000495. In red are highlighted peaks removed from the dataset because of their high frequencies. In green, peaks removed due to the strong deviation from the peptide mass cluster centres.

Figure 10 visualizes the result of non-peptide peak filtering in case of a dataset of 380 calibrated peak-lists. Spots removed by applying the filtering criterion Δλppm
 MathType@MTEF@5@5@+=feaafiart1ev1aaatCvAUfKttLearuWrP9MDH5MBPbIqV92AaeXatLxBI9gBaebbnrfifHhDYfgasaacH8akY=wiFfYdH8Gipec8Eeeu0xXdbba9frFj0=OqFfea0dXdd9vqai=hGuQ8kuc9pgc9s8qqaq=dirpe0xb9q8qiLsFr0=vr0=vr0dc8meaabaqaciaacaGaaeqabaqabeGadaaakeaacqGHuoardaqhaaWcbaacciGae83UdWgabaGaemiCaaNaemiCaaNaemyBa0gaaaaa@3430@(*m*, 0) > 200 are shown in green. Peptide masses removed due to filtering of abundant masses [12] are shown in red.

We studied how the non-peptide peak filtering influences the Probability Based Mascot Score (PBMS) [22]. In theory, for example one cystein rich peptide strongly deviating from the peptide mass rule and with a unique mass in the database digest, if properly assigned is sufficient to identify the protein unambiguously [10]. In case of PBMS, which requires multiple matches to peptide masses, a single match of a unique peptide mass, even if properly assigned, will not give a score indicating reliable identification of the protein. Furthermore, this scoring scheme takes into account the number of non-matching peaks. If many unassigned peaks are observed, the score is decreased and the assignment is interpreted as insignificant. Therefore, the removal of non-peptide peaks should increase the identification sensitivity. Table 4 demonstrates that an increase of 2.5% in the number of identified samples can be obtained by removing all peaks with a distance Δλppm
 MathType@MTEF@5@5@+=feaafiart1ev1aaatCvAUfKttLearuWrP9MDH5MBPbIqV92AaeXatLxBI9gBaebbnrfifHhDYfgasaacH8akY=wiFfYdH8Gipec8Eeeu0xXdbba9frFj0=OqFfea0dXdd9vqai=hGuQ8kuc9pgc9s8qqaq=dirpe0xb9q8qiLsFr0=vr0=vr0dc8meaabaqaciaacaGaaeqabaqabeGadaaakeaacqGHuoardaqhaaWcbaacciGae83UdWgabaGaemiCaaNaemiCaaNaemyBa0gaaaaa@3430@(*m*, 0) > 200*ppm *from the peptide peak-lists. Row 8 of Table 4 shows that non-peptide peak filtering increases the PBMS score in 30 – 55% of cases. Removal of peptide peaks due to filtering caused a decrease of the PBMS score in less than 1% of samples.

**Table 4:** Results for filtering of non-peptide masses.

		*Arabidopsis t*.	*Rhodopirelulla b*.	*Mus musculus*
1	Identification no *PR *filtering	423	1009	872
2	Identification with *PR *filtering	432	1017	894
3	Change in identification (Percent)	2.13	0.79	2.52

4	Total nr. of samples*	818	1169	1709
5	Nr. samples with PBMS increase	240	622	724
6	Nr. samples with no change of PBMS	571	542	982
7	Nr. samples with PBMS decrease	7	5	3
8	Percent increase of PBMS score	29.34	53.21	42.36
9	Percent decrease of PBMS score	0.86	0.43	0.18

Columns: *Arabidopsis t., Rhodopirelulla b., Mus musculus *– peptide mass fingerprint datasets (cf. Methods). Row 1 – number of samples with a significant PBMS score prior to filtering of non-peptide peak masses. Row 2 – number of samples with a significant PBMS score for peak-lists with non-peptide removed. Row 3 – relative change of the identification rate (Row 2 – Row 1)/Row1 100. Row 4 – Total number of samples which produced a PBMS score. Row 5 -number of samples for which an increase of the PBMS score due to non peptide peak filtering was observed. Row 6 – number of samples for which no change of the PBMS score due to non-peptide peak filtering was observed. Row 7 – number of samples for which a decrease of the PBMS score due to non-peptide peak filtering was observed. Row 8–9 – relative increase and decrease of the PBMS score, respectively.

We concluded that non-peptide peak filtering increases the sensitivity of protein identification if using the PBMS scoring schema. However, to which extend these results can be reproduced is dependent on the database search algorithm used.

## Conclusion

We introduced here a simple model to predict the cluster centres of peptide masses. The input parameters of the model can be easily determined for the sequence databases. We studied how these parameters influence the location of cluster centres, concluding that the cleavage specificity of the enzyme used for peptide digestion and the cleavage probability are the main factors. The change of the cluster centre location due to changes in average protein length or due to variability of amino acid frequencies among the databases is relatively small. However, our analysis also illustrates that, due to combinatorial constraints, the location of the cluster centres for masses smaller than l000*Da *can differ from the average location. Based on the model of the peptide mass cluster centres we derived a measure to determine the deviation of an experimental peptide mass from the nearest cluster centre. We used this distance measure to calibrate the peptide peak-lists and to recognise non-peptide peaks. The calibration method, linear regression on peptide rule, is a robust and accurate method to calibrate single peak lists without resorting to internal calibrants. With this method higher calibration precision was obtained in comparison to other calibration methods, which also employ the property of peptide mass clustering.

The same distance measure was used to recognise non-peptide peaks and to remove them from the peak-lists. Due to their removal, an increase of the identification rate of up to 2.5% for the PBMS scoring schema was observed.

## Methods

### Data sets

In this study, we used three data sets generated in different proteome analyses:

1. A bacterial proteome of *Rhodopirellula baltica *(unpublished data) (1,193 spectra) measured on a Reflex III [31] MALDI-TOF instrument.

2. A mammalian proteome of *Mus musclus *(1, 882 spectra) measured on an Ultraflex [31] MALDI-TOF instrument.

3. A plant proteome of *Arabidopsis thaliana *[32] measured on an Autoflex [31] MALDI-TOF instrument.

All PMF MS spectra derive from tryptic protein digests of individually excised protein spots. For this purpose, the whole tissue/cell protein extracts of the aforementioned organisms were separated by two-dimensional (2D) gel electrophoresis [33] and visualised with MS compatible Coomassie brilliant blue G250 [32]. The MALDI-TOF MS analysis was performed using a delayed ion extraction and by employing the MALDI AnchorChip ™targets (Bruker Daltonics, Bremen, Germany). Positively charged ions in the m/z range of 700 – 4, 500*m*/*z *were recorded. Subsequently, the SNAP algorithm of the XTOF spectrum analysis software (Bruker Daltonics, Bremen, Germany) detected the monoisotopic masses of the measured peptides. The sum of the detected monoisotopic masses constitutes the raw peak-list.

### Calibration

In order to perform filtering of non-peptide peaks the dataset must be calibrated to high mass measurement accuracy. To align the dataset we used a calibration sequence [12] consisting of several calibration procedures.

First calibration using external calibration samples was performed in order to remove higher order terms of the mass measurement error [11]. Next, the affine mass measurement error of all samples on the sample support was determined by linear regression on the peptide mass rule introduced here. Subsequently, the thin plate splines were used to model the mass measurement error in dependence of the sample support positions to calibrate the spectra. Finally, the spectra were aligned using a modified spanning tree algorithm [12].

### Mascot database search

Processed peak-lists were then used for the protein database searches with the Mascot search software (Version 1.8.1) [22], employing a mass accuracy of ± 0.1*Da*. Methionine oxidation was set as a variable and carbamidomethylation of cysteine residues as fixed modification. We allowed only one missed proteolytic cleavage site in the analysis.

### Sequence databases

We determined the amino acid frequencies of the nine protein sequence databases listed in Table 5. Seven of these databases are organism specific subsets of the *NCBI *non-redundant protein database [34].

**Table 5:** Protein lengths and amino acid frequencies (one letter code) for nine in the nine databases, *length *– average protein length in database, *reference *– database reference; *f*_*i *_– amino acid frequencies

Organ izm	*length*	*f*_*F*_	*f*_*S*_	*f*_*T*_	*f*_*N*_	*f*_*K*_	*f*_*Y*_	*f*_*E*_	*f*_*V*_	*f*_*Q*_	*f*_*M*_
*Arabidopsis t*.	422.40	4.27	9.01	5.11	4.41	6.36	2.86	6.74	6.69	3.52	2.44
*Drosophila m*.	506.20	3.48	8.33	5.68	4.80	5.70	2.91	6.41	5.88	5.21	2.33
*Escherichia coli*	300.30	3.86	6.25	5.67	4.26	4.59	2.96	5.65	6.91	4.40	2.67
*Homo sapiens*	360.40	3.61	8.61	5.55	3.55	5.54	2.86	6.81	6.02	4.80	2.12
*Mus musculus*	378.30	3.74	8.58	5.55	3.59	5.71	2.88	6.75	6.11	4.74	2.22
*Rattus norvegicus*	484.40	3.81	8.33	5.52	3.59	5.62	2.74	6.77	6.32	4.64	2.28
*Saccharomyces c*.	447.00	4.47	9.02	5.93	6.18	7.26	3.41	6.43	5.58	3.94	2.10
*Rhodopirellula b*.	314.70	3.70	7.37	5.85	3.37	3.44	2.09	6.02	7.05	4.04	2.43
SwissProt DB	367.90	4.03	6.89	5.47	4.22	5.93	3.09	6.59	6.70	3.93	2.38

Mean	397.96	3.89	8.04	5.59	4.22	5.57	2.87	6.46	6.36	4.36	2.33
SD	71.90	0.32	0.98	0.24	0.88	1.07	0.35	0.39	0.50	0.54	0.18
Min	300.30	3.48	6.25	5.11	3.37	3.44	2.09	5.65	5.58	3.52	2.10
Max	506.20	4.47	9.02	5.93	6.18	7.26	3.41	6.81	7.05	5.21	2.67

	*reference*	*f*_*C*_	*f*_*L*_	*f*_*A*_	*f*_*W*_	*f*_*P*_	*f*_*H*_	*f*_*D*_	*f*_*R*_	*f*_*I*_	*f*_*G*_

*Arabidopsis t*.	[34]	1.80	9.52	6.36	1.26	4.80	2.28	5.43	5.39	5.34	6.41
*Drosophila m*.	[34]	1.95	9.02	7.36	1.00	5.46	2.64	5.18	5.53	4.96	6.17
*Escherichia coli*	[34]	1.17	10.23	9.27	1.50	4.32	2.22	5.21	5.54	5.94	7.38
*Homo sapiens*	[34]	2.24	9.78	6.98	1.35	6.22	2.51	4.73	5.64	4.28	6.80
*Mus musculus*	[34]	2.29	9.92	6.86	1.29	6.03	2.57	4.76	5.51	4.38	6.54
*Rattus norvegicus*	[34]	2.29	10.07	6.88	1.25	5.97	2.58	4.77	5.59	4.51	6.49
*Saccharomyces c*.	[34]	1.30	9.52	5.51	1.04	4.39	2.18	5.76	4.41	6.58	5.00
*Rhodopirellula b*.	[37]	1.27	9.31	9.25	1.54	5.33	2.31	6.23	6.96	4.95	7.48
SwissProt	[27]	1.57	9.63	7.80	1.17	4.86	2.27	5.30	5.29	5.92	6.94

Mean		1.76	9.67	7.36	1.27	5.26	2.40	5.26	5.54	5.21	6.58
SD		0.45	0.38	1.25	0.18	0.71	0.18	0.50	0.65	0.80	0.74
Min		1.17	9.02	5.51	1.00	4.32	2.18	4.73	4.41	4.28	5.00
Max		2.29	10.23	9.27	1.54	6.22	2.64	6.23	6.96	6.58	7.48

### In silico protein digestion

The theoretical digestion of the protein databases was done with ProtDigest [35], a command line program taking a protein sequence database file in *fasta *format and cleavage specificities as input. Other optional input parameters included fixed as well as variable modifications and number of missed cleavages. The output file contains all theoretically resulting peptides with their corresponding masses.

### Regression analysis

The complete tryptic *insilico *digest of the SwissProt [27] database generated more than 7 million peptides. In order to compute the slope coefficient we were sampling 500 times 10000 monoisotopic and corresponding nominal masses. For each sample we fitted the affine linear model with and without fixed intercept using linear regression. The slope and intercept coefficients in Figure 1 are the medians of these 500 samples.

## Appendix

### Wool and Smilanskys algorithm

Wool and Smilansky [10] use a Discrete Fourier Transform (DFT) to determine the calibration coefficients. The wavelength *λ *of a peptide peak-list can be determined by convolution. The "time domain" is the peak-list *X *with masses *x*_*i*_. We computed the amplitude *A *(Equation 36) for a small range of frequencies (*ω *~ *f *= 1/*λ *around *λ*_*theo*_. We scanned the range *λ *∈ *λ*_*theo *_± 0.0005 in steps of 5·10^-7 ^computing, for each *λ*, the real part (Equation 35), the imaginary part (Equation 34) and the amplitude *A*(*ω*) (Equation 36):

*f *= 1/*λ ω *= 2*πf*,     (33)

ℑ(ω)=∑isin(ωxi),     (34)
 MathType@MTEF@5@5@+=feaafiart1ev1aaatCvAUfKttLearuWrP9MDH5MBPbIqV92AaeXatLxBI9gBaebbnrfifHhDYfgasaacH8akY=wiFfYdH8Gipec8Eeeu0xXdbba9frFj0=OqFfea0dXdd9vqai=hGuQ8kuc9pgc9s8qqaq=dirpe0xb9q8qiLsFr0=vr0=vr0dc8meaabaqaciaacaGaaeqabaqabeGadaaakeaaiiaacqWFresWdaqadiqaaGGaciab+L8a3bGaayjkaiaawMcaaiabg2da9maaqafabaacbiGae03CamNae0xAaKMae0NBa42aaeWaceaacqGFjpWDcqWG4baEdaWgaaWcbaGaemyAaKgabeaaaOGaayjkaiaawMcaaiabcYcaSaWcbaGaemyAaKgabeqdcqGHris5aOGaaCzcaiaaxMaadaqadiqaaiabiodaZiabisda0aGaayjkaiaawMcaaaaa@4637@

ℜ(ω)=∑icos(ωxi),     (35)
 MathType@MTEF@5@5@+=feaafiart1ev1aaatCvAUfKttLearuWrP9MDH5MBPbIqV92AaeXatLxBI9gBaebbnrfifHhDYfgasaacH8akY=wiFfYdH8Gipec8Eeeu0xXdbba9frFj0=OqFfea0dXdd9vqai=hGuQ8kuc9pgc9s8qqaq=dirpe0xb9q8qiLsFr0=vr0=vr0dc8meaabaqaciaacaGaaeqabaqabeGadaaakeaaiiaacqWFCeIWdaqadiqaaGGaciab+L8a3bGaayjkaiaawMcaaiabg2da9maaqafabaacbiGae03yamMae03Ba8Mae03Cam3aaeWaceaacqGFjpWDcqWG4baEdaWgaaWcbaGaemyAaKgabeaaaOGaayjkaiaawMcaaiabcYcaSaWcbaGaemyAaKgabeqdcqGHris5aOGaaCzcaiaaxMaadaqadiqaaiabiodaZiabiwda1aGaayjkaiaawMcaaaaa@463B@

A(ω)=ℑ(ω)2+ℜ(ω)2.     (36)
 MathType@MTEF@5@5@+=feaafiart1ev1aaatCvAUfKttLearuWrP9MDH5MBPbIqV92AaeXatLxBI9gBaebbnrfifHhDYfgasaacH8akY=wiFfYdH8Gipec8Eeeu0xXdbba9frFj0=OqFfea0dXdd9vqai=hGuQ8kuc9pgc9s8qqaq=dirpe0xb9q8qiLsFr0=vr0=vr0dc8meaabaqaciaacaGaaeqabaqabeGadaaakeaacqWGbbqqdaqadiqaaGGaciab=L8a3bGaayjkaiaawMcaaiabg2da9maakaaabaGaeyyeHe8aaeWaceaacqWFjpWDaiaawIcacaGLPaaadaahaaWcbeqaaiabikdaYaaakiabgUcaRiabgYricpaabmGabaGae8xYdChacaGLOaGaayzkaaWaaWbaaSqabeaacqaIYaGmaaaabeaakiabc6caUiaaxMaacaWLjaWaaeWaceaacqaIZaWmcqaI2aGnaiaawIcacaGLPaaaaaa@44B1@

The wavelength of the masses in the peak-list is the *λ *at the maximum of *A*(*ω*). The phase for this *ω*_0 _= *ω*_max *A*(*ω*) _can be determined by:

ϕ0=ϕ(ωmax⁡A(ω))=arctan⁡(ℑ(ω0)2ℜ(ω0)2)⋅     (37)
 MathType@MTEF@5@5@+=feaafiart1ev1aaatCvAUfKttLearuWrP9MDH5MBPbIqV92AaeXatLxBI9gBaebbnrfifHhDYfgasaacH8akY=wiFfYdH8Gipec8Eeeu0xXdbba9frFj0=OqFfea0dXdd9vqai=hGuQ8kuc9pgc9s8qqaq=dirpe0xb9q8qiLsFr0=vr0=vr0dc8meaabaqaciaacaGaaeqabaqabeGadaaakeaaiiGacqWFvpGAdaWgaaWcbaGae8hmaadabeaakiabg2da9iab=v9aQnaabmGabaGae8xYdC3aaSbaaSqaaiGbc2gaTjabcggaHjabcIha4jabdgeabnaabmGabaGae8xYdChacaGLOaGaayzkaaaabeaaaOGaayjkaiaawMcaaiabg2da9iGbcggaHjabckhaYjabcogaJjabcsha0jabcggaHjabc6gaUnaabmGabaWaaSaaaeaacqGHresWdaqadiqaaiab=L8a3naaBaaaleaacqaIWaamaeqaaaGccaGLOaGaayzkaaWaaWbaaSqabeaacqaIYaGmaaaakeaacqGHCeIWdaqadiqaaiab=L8a3naaBaaaleaacqaIWaamaeqaaaGccaGLOaGaayzkaaWaaWbaaSqabeaacqaIYaGmaaaaaaGccaGLOaGaayzkaaGaeyyXICTaaCzcaiaaxMaadaqadiqaaiabiodaZiabiEda3aGaayjkaiaawMcaaaaa@5E8C@

The peak centres are at the line:

M′=2⋅πω0⋅N+ϕ0ω0  where  N=1,2,...,n.     (38)
 MathType@MTEF@5@5@+=feaafiart1ev1aaatCvAUfKttLearuWrP9MDH5MBPbIqV92AaeXatLxBI9gBaebbnrfifHhDYfgasaacH8akY=wiFfYdH8Gipec8Eeeu0xXdbba9frFj0=OqFfea0dXdd9vqai=hGuQ8kuc9pgc9s8qqaq=dirpe0xb9q8qiLsFr0=vr0=vr0dc8meaabaqaciaacaGaaeqabaqabeGadaaakeaadaWfGaqaaiabd2eanbWcbeqaaGGaaiab=jdiIcaakiabg2da9maalaaabaGaeGOmaiJaeyyXICncciGae4hWdahabaGae4xYdC3aaSbaaSqaaiabicdaWaqabaaaaOGaeyyXICTaemOta4Kaey4kaSYaaSaaaeaacqGFvpGAdaWgaaWcbaGaeGimaadabeaaaOqaaiab+L8a3naaBaaaleaacqaIWaamaeqaaaaakiabbccaGiabbccaGiabbEha3jabbIgaOjabbwgaLjabbkhaYjabbwgaLjabbccaGiabbccaGiabd6eaojabg2da9iabigdaXiabcYcaSiabikdaYiabcYcaSiabc6caUiabc6caUiabc6caUiabcYcaSiabd6gaUjabc6caUiaaxMaacaWLjaWaaeWaceaacqaIZaWmcqaI4aaoaiaawIcacaGLPaaaaaa@5D33@

But they should be on the line:

*M *= *λ*_theo _* *N*.         (39)

Solving Equation 38 for N and substituting N in the Equation 39 yields the Equation:

M=λtheo⋅ω02⋅π(M′−ϕ0ω0),     (40)
 MathType@MTEF@5@5@+=feaafiart1ev1aaatCvAUfKttLearuWrP9MDH5MBPbIqV92AaeXatLxBI9gBaebbnrfifHhDYfgasaacH8akY=wiFfYdH8Gipec8Eeeu0xXdbba9frFj0=OqFfea0dXdd9vqai=hGuQ8kuc9pgc9s8qqaq=dirpe0xb9q8qiLsFr0=vr0=vr0dc8meaabaqaciaacaGaaeqabaqabeGadaaakeaacqWGnbqtcqGH9aqpdaWcaaqaaGGaciab=T7aSnaaBaaaleaacqWG0baDcqWGObaAcqWGLbqzcqWGVbWBaeqaaOGaeyyXICTae8xYdC3aaSbaaSqaaiabicdaWaqabaaakeaacqaIYaGmcqGHflY1cqWFapaCaaGaeiikaGYaaCbiaeaacqWGnbqtaSqabeaaiiaacqGFYaIOaaGccqGHsisldaWcaaqaaiab=v9aQnaaBaaaleaacqaIWaamaeqaaaGcbaGae8xYdC3aaSbaaSqaaiabicdaWaqabaaaaOGaeiykaKIaeiilaWIaaCzcaiaaxMaadaqadiqaaiabisda0iabicdaWaGaayjkaiaawMcaaaaa@5195@

α=λtheo⋅ω02⋅π and β=ϕ0ω0 and(41)mcorr=α(mexp−β)=αmexp−αβ,     (42)
 MathType@MTEF@5@5@+=feaafiart1ev1aaatCvAUfKttLearuWrP9MDH5MBPbIqV92AaeXatLxBI9gBaebbnrfifHhDYfgasaacH8akY=wiFfYdH8Gipec8Eeeu0xXdbba9frFj0=OqFfea0dXdd9vqai=hGuQ8kuc9pgc9s8qqaq=dirpe0xb9q8qiLsFr0=vr0=vr0dc8meaabaqaciaacaGaaeqabaqabeGadaaakeaafaqadeGacaaabaacciGamaiGy6paa8xSdeMamaiGy6paayypa0ZaiaiGy6paaSaaaeacaciM+daacWaGaIP=aaWF7oaBdGaGaIP=aaWgaaWcbGaGaIP=aaGamaiGy6paamiDaqNamaiGy6paamiAaGMamaiGy6paamyzauMamaiGy6paam4Ba8gabKaGaIP=aaaakiadaciM+daagwSixladaciM+daa=L8a3nacaciM+daaBaaaleacaciM+daacWaGaIP=aaaIWaamaeqcaciM+daaaaGcbGaGaIP=aaGamaiGy6paaGOmaiJamaiGy6paayyXICTamaiGy6paa8hWdahaaiadaciM+daabccaGiadaciM+daabggaHjadaciM+daab6gaUjadaciM+daabsgaKjadaciM+daabccaGiadaciM+daa=j7aIjadaciM+daag2da9macaciM+daalaaabGaGaIP=aaGamaiGy6paa8x1dO2aiaiGy6paaSbaaSqaiaiGy6paaiadaciM+daaicdaWaqajaiGy6paaaaakeacaciM+daacWaGaIP=aaWFjpWDdGaGaIP=aaWgaaWcbGaGaIP=aaGamaiGy6paaGimaadabKaGaIP=aaaaaaGccWaGaIP=aaqGGaaicWaGaIP=aaqGHbqycWaGaIP=aaqGUbGBcWaGaIP=aaqGKbazaeaadaqadiqaaiabisda0iabigdaXaGaayjkaiaawMcaaaqaaiabd2gaTnaaBaaaleaacqWGJbWycqWGVbWBcqWGYbGCcqWGYbGCaeqaaOGaeyypa0Jae8xSdeMaeiikaGIaemyBa02aaSbaaSqaaGqaciab+vgaLjab+Hha4jab+bhaWbqabaGccqGHsislcqWFYoGycqGGPaqkcqGH9aqpcqWFXoqycqWGTbqBdaWgaaWcbaGae4xzauMae4hEaGNae4hCaahabeaakiabgkHiTiab=f7aHjab=j7aIjabcYcaSiaaxMaacaWLjaaabaWaaeWaceaacqaI0aancqaIYaGmaiaawIcacaGLPaaaaaaaaa@F59D@

*m*_*corr *_= *α*(*m*_*exp *_- *β*) = *αm*_*exp *_- *αβ*,     (42)

which can be used to correct the masses. This is an affine linear model with two coefficients *α *and *αβ*.

## Abbreviation

• PBMS – Probability based Mascot score

• DFT – Discrete Fourier Transformation

• *m*/*z *– mass over charge

## Authors' contributions

WEW developed and implemented the methods described, carried out the analysis and visualised the results.

WEW, MF, ML and AKE wrote the manuscript.

AKE implemented the sequence digester.

All authors contributed to the final version of the manuscript.
